# Stage-specific differential gene expression in *Leishmania infantum*: from the foregut of *Phlebotomus perniciosus* to the human phagocyte

**DOI:** 10.1186/1471-2164-15-849

**Published:** 2014-10-03

**Authors:** Pedro J Alcolea, Ana Alonso, Manuel J Gómez, Marina Postigo, Ricardo Molina, Maribel Jiménez, Vicente Larraga

**Affiliations:** Laboratorio de Parasitología Molecular, Departamento de Microbiología Molecular y Biología de las Infecciones, Centro de Investigaciones Biologicas, Consejo Superior de Investigaciones Científicas, Calle Ramiro de Maeztu, 9, 28040 Madrid, Spain; Unidad de Secuenciación y Bioinformática, Centro de Astrobiología, Instituto Nacional de Técnica Aeroespacial “Esteban Terradas” and Consejo Superior de Investigaciones Científicas, Ctra. Ajalvir Km 4., 28850 Torrejón de Ardoz, Spain; Unidad de Entomología Médica, Servicio de Parasitología, Centro Nacional de Microbiología, Instituto de Salud Carlos III, Ctra. Majadahonda-Pozuelo s/n, 28220 Majadahonda, Spain; Centro Nacional de Investigaciones Cardiovasculares, Madrid, Spain

**Keywords:** *Leishmania infantum*, *Phlebotomus perniciosus*, Promastigotes, Amastigotes, Promastigote axenic culture, Gene expression profiling

## Abstract

**Background:**

*Leishmania infantum* is the etiological agent of zoonotical visceral leishmaniasis in the Mediterranean basin. A recent outbreak in humans has been recently reported in central Spain. *Leishmania* spp. parasites are transmitted to the mammalian host by the bite of sand flies. The primary vector of *L. infantum* in Spain is *Phlebotomus perniciosus*. For decades, research on these parasites has involved the axenic culture model of the promastigote stage including gene expression profiling studies performed in the post-genome era. Unlike the controversial axenic culturing of amastigotes, promastigote cultures are generally accepted and used, although with the precaution of avoiding excessive culture passage.

The primary objective of this differentiation study is to compare the gene expression profiles of promastigotes isolated from the foregut of the sand fly and amastigotes. For this purpose, *P. perniciosus* sand flies were infected with *L. infantum* and differentiated promastigotes were extracted by dissection of the foreguts. Shotgun DNA microarray hybridization analyses allowed for transcriptome comparison of these promastigotes with amastigotes obtained by infection of the U937 cell line. The results have been compared with those described in published expression analyses using axenic promastigotes.

**Results:**

A total of 277 up-regulated genes were found through this hybridization experiment. The comparison of these particular results with published gene expression profile analyses performed using the same experimental procedure to study cultured promastigotes in stationary phase *versus* amastigotes revealed considerable differences (approximately 95% of the up-regulated genes were different). We found that the up-regulation rate is lower in amastigotes than in sand fly-derived promastigotes, which is in agreement with the over-expression of genes involved in gene expression regulation and signaling in those promastigote populations.

**Conclusions:**

The up-regulation rate is lower in intracellular amastigotes than in promastigotes obtained from the sand fly gut. This was also reported by us using the promastigote culture model and is an evidence for the hypothesis of promastigote preadaptation towards life in the intracellular environment. Regarding transcript abundance, the set of differentially regulated genes is notably different when using promastigotes from the sand fly foregut instead of axenic cultures.

**Electronic supplementary material:**

The online version of this article (doi:10.1186/1471-2164-15-849) contains supplementary material, which is available to authorized users.

## Background

**Figure 1:**
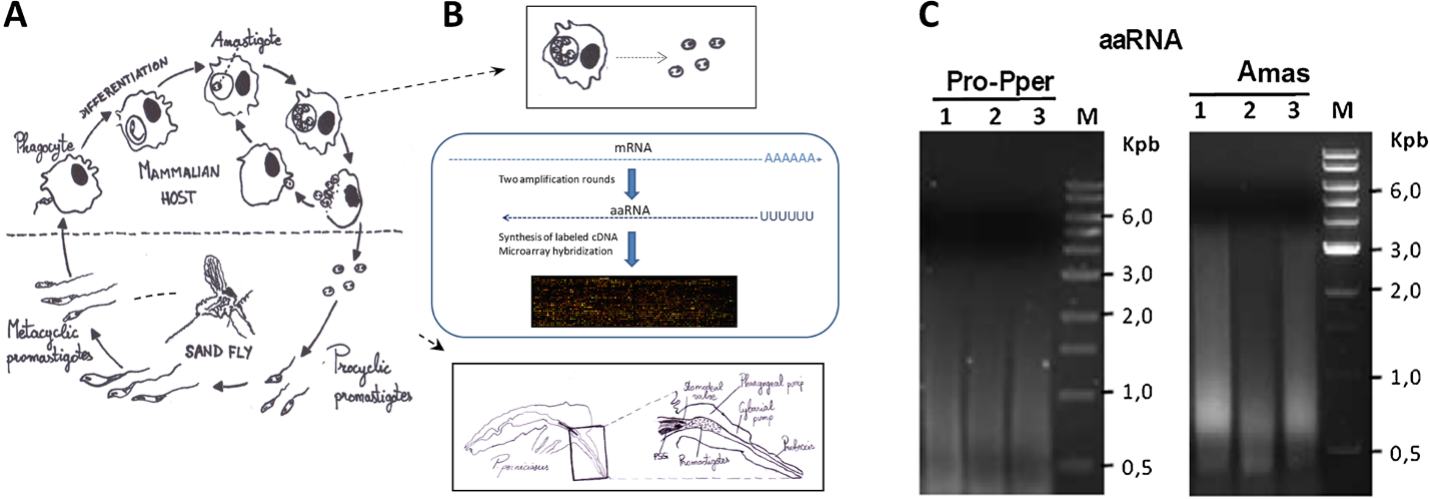
**Sampling and mRNA amplification. (A)** The life cycle of *Leishmania* spp. **(B)** Promastigote RNA extraction was performed immediately after dissection of the sand fly guts and mild lysis of U937 cells. Pro-Pper samples were immediately washed in PBS and lysed with TRIzol® for total RNA extraction. After that, mRNA was doubly amplified (aaRNA) due to sample amount requirements. This included two cycles of reverse transcription (RT) plus second strand cDNA synthesis (combining the use of the Klenow fragment and the RNase H) plus *in vitro* transcription (IVT). The RT reaction of the first amplification round was performed with a poly-dT primer and the second strand synthesis and the RT reaction of the second amplification round were performed with random hexamer primers, all of which were provided in the MessageAmp^TM^ II aRNA Amplification Kit. Three biological replicates were obtained to perform the subsequent microarray experiment. **(C)** Electrophoresed aaRNA samples used for the microarray analysis after synthesis of labeled cDNA.

Leishmaniasis is a compendium of neglected vector-borne infectious diseases caused by kinetoplastid protozoa of the genus *Leishmania* with an estimated prevalence of 12 million people worldwide. Visceral leishmaniasis is fatal without treatment and annually leads to 60,000 deaths at least [1, 2]. *L. infantum* is the ethiological agent of zoonotic visceral leishmaniasis in the Mediterranean basin and this species also acts as an opportunistic pathogen, as indicated by the increase in co-infections with HIV [3, 4]. An important outbreak of human leishmaniasis has been reported recently in Fuenlabrada, located in the southwest of the Madrid region [5, 6]. The life cycle of the parasite (Figure 1A) is dimorphic and digenetic because the two stages develop in different hosts. Procyclic promastigotes differentiate to metacyclics inside the gut of female sand flies (Diptera: Psychodidae, Phlebotominae), which inject parasites into the mammalian host during blood feeds. Amastigotes survive inside parasitophorous vacuoles of phagocytic mononuclear cells and are able to infect other phagocytes after subsequent proliferation. *Phlebotomus perniciosus* and *P. ariasi* are the proven vectors of *L. infantum* in Spain [7] and *P. perniciosus* is the major vector of *L. infantum* in the central and western Mediterranean basin [8].

The difficulty of studying promastigotes in their natural environment, the gut of the sand fly, is due to manipulation and reduced biomass. To overcome these problems, axenic cultures of *Leishmania* spp. promastigotes in liquid media were developed in the 1960s and 70s in an attempt to reproduce *in vitro* the conditions inside the gut of the sand fly [9–12]. These media are undefined, as they contain macromolecules, proteins, lipoid substances, trace elements and low molecular weight nutrients. Promastigote cultures are incubated generally at approximately 26–27°C also imitating the conditions inside the gut of the sand fly (reviewed by [13, 14]). Promastigotes are maintained in culture for over a week reaching stationary phase and then the death phase, although a considerable proportion of the cells are able to survive for weeks. Compared with the axenic culture model of amastigotes [15, 16], the promastigote culture model is stable and reproducible and is widely used for the study of almost all aspects of the biology of this stage in all species of the genus *Leishmania*. In fact, it is used so routinely that the status of axenically cultured promastigotes has been rarely considered. However, it has been reported that after numerous passages, the original features, infectivity and virulence of the parasite become attenuated, and they frequently require passages through laboratory animals, such as hamsters (reviewed in [13]). Culture passaging does not affect structural studies on specific proteins, as an example, but other research may be affected, such as the evaluation of infectivity, parasite-host cell interactions or the immune response of the host.

The analysis of stage-specific gene regulation in trypanosomatids has provided not only data about the particular expression profiles of hundreds of genes but also valuable information about the biology of these pathogens. First, low stage-specific regulation rates have been described [15–29]. Furthermore, expression profiling confirms that axenically cultured amastigotes are not equivalent to intracellular amastigotes in *L. mexicana* [21] and in *L. infantum* [15, 16]. Saxena et al. [19] reported that differentiation of *L. donovani* promastigotes to amastigotes is achieved by a succession of transient and permanent changes in gene expression. In addition, we described the up-regulation of genes directly and indirectly related to infectivity in metacyclic PNA^-^ promastigotes in *L. infantum* [26], found a lower up-regulation rate in amastigotes with respect to promastigotes [23] and more relevance of temperature increase than acidification in the differentiation process of promastigotes to amastigotes, as well as the confluence of both factors leading to an amastigote-like profile [16].

Even though a limited amount of RNA from promastigotes from the sand fly gut anterior to the stomodeal valve can be isolated, a possibility to overcome this limitation is mRNA amplification. However, the small amount of protein extract from this kind of biological samples would not allow performing proteome analyses with the current approaches. Bearing this in mind, we compared the stage-specific gene expression of metacyclic promastigotes and amastigotes in their natural environments for the first time using a high-throughput transcriptome analysis, which revealed noticeable differences between the expression profiles of uncultured and cultured promastigotes when compared to amastigotes.

## Methods

### Promastigote culture, *in vitro*infection of phagocytes and amastigote isolation

The *Leishmania infantum* isolate MCAN/ES/98/10445 (zymodeme MON-1) was cultured in complete medium containing RPMI 1640 supplemented with L-glutamine (Cambrex, Karlskoga, Sweden), 10% heat inactivated fetal bovine serum (HIFBS) (Cambrex) and 100 μg/ml streptomycin – 100 IU/ml penicillin (Cambrex) at 27°C. They were used in passages 5 to 10 after extraction of the sand fly foregut to perform *in vitro* infections (see below) of the U937 cell line from human histiocytic leukemia (ATCC® CRL1593.2) [30] and again to feed sand flies to collect metacyclic promastigote samples from the foregut for the microarray analysis. Both sand fly infection steps were carried out following the procedure detailed in the next subsection. In the first case, promastigotes recovered from the foregut were established in NNN medium and subsequently in complete medium until the specified number of passages. Stationary phase promastigotes were harvested at 2000 g for 10 min.

Cultures of the U937 cell line were carried out at 37°C, 5% CO_2_ in complete medium for 72 h and centrifuged at 250 g, followed by a 72 h incubation in complete medium with 20 ng/ml phorbol 12-myristate 13-acetate (Sigma, Saint Louis, MO) for stimulation [31]. Adhered cells were mildly rinsed with RPMI supplemented with L-glutamine (Cambrex) and recovered by vigorous shaking and in the presence of 0.5 g/l trypsin and 0.2 g/l EDTA (Cambrex). Trypsin was inactivated with 1 volume of complete medium and phagocytes were harvested. Infections were performed by incubating 20 × 10^6^ promastigotes/ml: 10^6^ macrophages/ml at 37°C for 2 h in complete medium in a water bath and mild shaking every 15 min. After that, the mixture was centrifuged at 250 g for 10 min and incubated in complete medium at 37°C, 5% CO_2_ for 72 h. After 2 and 16 h, the cultures were rinsed with complete medium. Once phagocytes were detached again, amastigotes were isolated by mild lysis of phagocytes with 0.5% SDS in RPMI with vigorous agitation for 1 min followed by centrifugation at 13,000 g for 1 min [32]. Aliquots of the amastigote suspension were checked by Giemsa stain and gp63/gp46 immunofluorescence analysis as previously described [23].

### Infection of *Phlebotomus perniciosus*and isolation of promastigotes

Infected U937 cells were rinsed and detached as described above. Next, they were resuspended at 10^6^ cells/ml in defibrinated rabbit blood. The mixture was used to feed 150–200 female sand flies of an established colony [33] over a 3-day chicken skin membrane. The sand flies were maintained in a climatic chamber at 27–28°C, 90-100% relative humidity, 17 h light / 7 h darkness photoperiod and 30% fructose solution. Promastigote morphology and location inside the gut of a subset of sand flies were evaluated daily by light microscopy. Metacyclic promastigotes anterior to the stomodeal valves (Pro-Pper) were recovered in PBS with a sterile Pasteur pipette [34] from the foregut at the proper times (5–7 days) depending on the previous observations and immediately centrifuged. For this purpose, dissection of the sand flies was performed for extraction of the digestive tracts, which were then split open by pressure with a coverslip. An aliquot was previously recovered for cell counting.

### RNA isolation, mRNA amplification and synthesis of labeled cDNA

Total RNA from three biological replicates of each condition was immediately extracted with TRizol® reagent (Life Technologies, Carlsbad, CA) following the manufacturer’s instructions. The volume of TRIzol® reagent used was 0.5 ml for each of three Pro-Pper replicates and 1 ml for amastigote samples. Glycogen at 1 μg/ml (Life Technologies) was used as carrier prior to 2-propanol precipitation in the total RNA isolation procedure of Pro-Pper samples. RNA quality was assessed with an Experion RNA HighSens Analysis Kit (Bio-Rad Laboratories, Hercules, CA) and conventional agarose gel electrophoresis. Thereafter, two mRNA amplification rounds were performed with MessageAmp^TM^ II aRNA Amplification Kit (Life Technologies) as previously described [23] thus yielding antisense doubly amplified RNA (aaRNA). The integrity of aRNA and aaRNA samples was checked by 1% agarose gel electrophoresis.

The first strand aminoallyl-cDNA was synthesized. First, denaturing of 10 μg of aaRNA together with 6 μg of random primers (Life Technologies) was carried out by incubation at 70°C for 10 min and snap-chill on ice. Then, samples were incubated at 46°C for 3 h with 570 μM each dATP, dCTP, dGTP, 230 μM dTTP, 340 μM aminoallyl-dUTP, 10 μM DTT and 600 U SuperScript® Reverse Transcriptase (Life Technologies) in a 30 μl final volume. Then, a 70°C, 30 min incubation in 100 mM NaOH/10 mM EDTA allowed DNA degradation. After neutralization with 3 μl of 3 M sodium acetate pH 5.2, single stranded cDNA samples were purified with QiaQuick PCR Purification Kit (Qiagen, Hilden, Germany) using phosphate wash buffer (5 mM KPO_4_, 80% ethanol, pH 8.0) and phosphate elution buffer (4 mM KPO_4_) instead of the wash and elution buffers provided in the kit. Next, samples were completely dried in a vacuum centrifuge and resuspended in 10 μl of water, mixed with 5 μl of 12 ng/μl DMSO-dissolved Cy3 or Cy5 monofunctional dye (respectively for amastigotes and promastigotes) (GE Healthcare, Chalfont Saint Giles, UK) and incubated at room temperature in darkness for 1 h for coupling with the aminoallyl residues. Labeled cDNA samples were then purified with a QiaQuick PCR Purification Kit (Qiagen) entirely following the manufacturer’s instructions.

### Microarray hybridization and analysis of data

The construction of the complete shotgun genomic DNA microarrays of *L. infantum* used has been published [26] and deposited in the GEO repository supplying MIAME compliant data (http://www.ncbi.nlm.nih.gov/geo/query/acc.cgi?acc=GSE11269). Prior to hybridization, the microarrays were soaked first in 0.1% N-lauroylsarcosine in 2x SSC, then soaked in 2x SSC and then denatured at 95°C for 3 min, fixed in chilled 100% ethanol and spun dry in a slide mini centrifuge. The microarrays were blocked by attachment upside down to a 60 ml drop of 3x SSC, 0.3% N-lauroylsarcosine, 60 mM Tris-HCl pH8.0, 83 ng/ml denatured herring sperm DNA and 1% BSA over a Hybri-Slip coverslip (Sigma) and incubated at 42°C in a water bath for 30 min. Then, labeled cDNA samples were mixed in equimolar amounts of each dye (50 pmol) and incubated at 40°C with blocked microarrays for 16 h (same composition of blocking solution except for 0.1% BSA, 25 ng/ml poly (T), 50% deionized formamide). After that, the slides were soaked in 2x SSC, 0.2% SDS at 40°C and consecutively in 1x SSC and 0.2x SSC at room temperature.

Genomic DNA was isolated from non-infected sand flies and U937 cells by phenolic extraction as described previously [26] and directly labeled with Cy5 (350 μM each dATP, dCTP, dGTP and (1/3 Cy5-dUTP, 2/3 dTTP) mix) using GenomiPhi^TM^ DNA Amplification Kit (GE Healthcare). Single dye hybridizations with *L. infantum* DNA microarrays were performed as a cross-hybridization control.

The hybridized slides were scanned with a GenePix 4100A instrument (Axon, Foster City, CA) and raw data with local feature background medians subtracted were obtained with GenePix Pro 7.0 software. Normalization with the LOWESS per pin algorithm and statistical inference using the paired t-test and FDR adjustment were performed with AlmaZen software (BioAlma, Tres Cantos, Spain) and checked with the TIGR Multi Experiment Viewer 4.3. The cutoff values were the following: (i) fold change F ≥ 2 (Cy5/Cy3 ratio if Cy5 > Cy3) or F ≤ -2 (-Cy3/Cy5 ratio if Cy3 > Cy5), (ii) total relative fluorescence intensity value > 5000 arbitrary fluorescence units and (iii) p* < 0.05. Three replicates were considered in the experiment.

### Identification of stage-regulated genes

The insert ends of clones that fulfilled the cutoff values mentioned were recovered from the genomic library used for microarray construction, sequenced with the M13-pUC18 primers and assembled as described, a strategy that is not affected by insertions, deletions and substitutions between the MCAN/ES/98/10445 and the genome-sequenced MCAN/ES/98/LLM-877 isolates [26]. The conditions used to consider the sequence of a given clone assembled were: (i) e-value < 1e-10 for both ends, (ii) convergent orientation in the genome sequence and (iii) length ≤ 11 kbp, according to the features of the genome library [26]. The analyzed clones were classified in three categories according to the fulfillment of such conditions: in *a* clones, only one pair of alignments complies with all three conditions; in *b* clones, more than one pair does due to adjoining sequence repeats and is therefore the best sequence identity; and *c* clones do not fulfill the requirements to be assembled for unpaired alignment or incongruent pair of alignments presumably due to the presence of two or more inserts in the clone. Once clones were assembled, identification of genes overlapping with them was performed using a Perl script with a 5% overlapping length cutoff. Clones that do not fulfill this criterion but align with less than 5% of the length of a given annotated ORF were identified using the genome browser [26]. Those clones that do not map with any ORF were aligned with complete transcript sequences including UTRs that were obtained by RNAseq in *L. major* [35]. Gene sequences were analyzed with BLAST2GO [36] to classify them in functional categories. In addition, the search of all genes in literature and the databases GeneDB [37], TriTrypDB [38] and KEGG [39] provided further functional information. CLUSTALW2 alignments allowed distinguishing gene copies from genes encoding isoforms.

### Real time quantitative RT-PCR (qRT-PCR) validation

Unlabelled single stranded cDNA was synthesized following the same procedure described above but using a mixture stock of 10 mM of each dNTP. Custom TaqMan® MGB Assays-by-Design (specifically FAM-NFQ MGB probes) (Life Technologies) were run in a 7900HT Fast Real Time PCR system (Life Technologies) using TaqMan® Universal Master Mix 2x (Life Technologies) following the manufacturer’s instructions. Thermal cycling was as follows: 95°C for 5 min, 40 cycles [95°C for 30”, 60°C for 1 min]. PCR efficiencies were calculated by the standard curve best fit method from a triplicate dilution series experiment for each gene and cDNA sample (Pro-Pper/Amastigotes). Coefficients of variation were previously checked. Fold changes were calculated with respective efficiency-corrected normalized quantities in the same fashion as for microarray data. Normalized quantities were calculated by dividing the raw quantity value (efficiency to the power of –Ct) of the gene of interest by that of the endogenous control (GAPDH gene of *L. infantum*). Sequences of primers and probes are listed in the Additional file 1.

### Binomial test and hierarchical clustering

A binomial test was performed to infer the level of significance of the differences in absolute frequencies of up-regulated and down-regulated genes in Pro-Pper/A as previously described [23]. An iterative hierarchical clustering analysis was also carried out with TIGR’s MultiExperiment Viewer 4.3 (MEV) by introducing normalized microarray hybridization data matrixes (including medians and standard deviations of intensity and F values) of clones with significant differential regulation in the experiment reported herein and the previously available data describing differential gene expression profiles of cultured amastigotes and amastigote-like forms [16, 23]. The SAM p-value cutoff was 0.05, which was the same as for the previous independent t-tests for each experiment. HCL-ST was performed independently for significant and non-significant genes. ST algorithm with a jackknifing resampling option and 100 iterations for the construction and clustering of the gene expression matrix were applied in a HCL-ST analysis.

## Results and discussion

### mRNA amplification and microarray hybridization analysis of metacyclic promastigotes isolated from *P. perniciosus*and amastigotes

**Figure 2:**
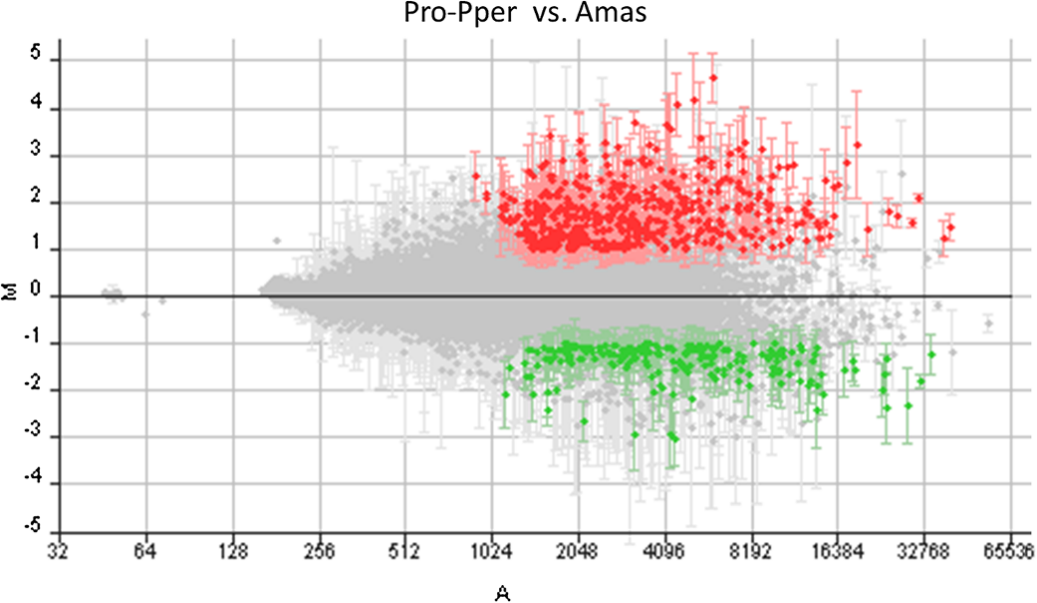
**General outcome of the Pro-Pper/A microarray hybridization experiment in** ***L. infantum*** **.** M/A scatter plot of hybridization outcomes of all clones fulfilling (highlighted) or not the conditions necessary for containing differentially regulated genes between Pro-Pper and Amas. M = (log_2_Ri – log_2_Gi) and A = [(log_2_Ri + log_2_Gi)/2], where R and G are, respectively, red (Cy5) and green (Cy3) fluorescence intensity values. Red spots correspond to selected DNA fragments containing a gene up-regulated by at least 2-fold and green spots represent those down-regulated by at least 2-fold times. Further criteria for spot selection are detailed in the Methods section.

The total amounts of RNA obtained from Pro-Pper replicates were comprised between 20 and 25 ng and after the first amplification round, 200–250 ng of aRNA were obtained. Double amplification of mRNA made the microarray hybridization experiments possible. Obviously RNA samples from amastigotes were treated identically. Electrophoretic analyses of the aaRNA samples including replicates are shown in Figure 1. The number of differences in gene expression found between Pro-Pper and amastigotes is 277 (Figure 2, Table 1), which is comparable to stage-specific gene expression regulation between logarithmic phase promastigotes and amastigotes and higher than between stationary phase promastigotes and amastigotes [23]. According to the 5% of clone-to-ORF overlapping length cutoff performed with a Perl script (see Methods section) [16, 23–26], 143 out of 277 differences correspond to genes of known function or hypothetical proteins genes. The 134 clones (48%) that do not fulfill this criterion (Table 1) are described in the Additional file 2. Some of them are aligned with less than 5% of the length of an ORF. The rest of clones do not align with any ORF but presumably do with untranslated regions (UTRs). For this reason, they were aligned against complete transcript sequences of *L. major* including UTRs that were obtained by RNAseq [35]. About half of the *Leishmania* spp. genes code for hypothetical proteins and proteins of unknown function [37, 38, 40, 41] and this is reflected in the relatively high number of such proteins that are differentially regulated (Table 1, Additional file 2). These facts enable the possibility of extracting additional information from the genome and the transcriptome of these parasites. Redundancy in representation of genome sequences by the genomic library generated for microarray construction [26] is reflected in stage-specific gene expression results because some clones represent the same differentially regulated gene (Table 2). This is an internal validation together with the control spots included in the microarrays [26] (Additional file 3).

**Table 1:** **Overview of the Pro-Pper/A differential gene expression profiles**

Annotation status	Frequency of differentially regulated genes in Pro-Pper/A	
	Up-regulated	Down-regulated
Genes of known function	46	19
Conserved hypothetical protein	48	25
Hypothetical protein	4	1
Clones overlapping with UTRs or less than 5% of an ORF (Additional file 2: Table S2)	86	48
Total (n = 277)	184	93

Absolute frequencies of genes encoding for proteins of known function and hypothetical proteins are provided. The frequencies of up-regulated and down-regulated genes have been contrasted with the binomial test.

**Table 2:** **Genes of known function that are differentially regulated in Pro-Pper with respect to amastigotes**

***Clone***	***F***	***Log***_***2***_ ***R ± S***	***p***	***e-value***		***Def.***	***Annotation***	***Annotated gene function***	***qRT-PCR***	
				***Fw***	***Rv***					
Lin13C3	2.72	1.4 ± 0.3	0.011	0	0	b	LinJ.21.0770	Ribonuclease-L inhibitor, ABC subfamily E, putative		N.D.
Lin16F1	3.69	1.9 ± 0.4	0.014	-	0	c	LinJ.23.0050	Peroxidoxin/tryparredoxin peroxidase		N.D.
Lin16C2	24.97	4.6 ± 0.5	0.004	0	0	b	LinJ.35.3930	EF-hand calmodulin-like protein	+	65.3 ± 3.1
							LinJ.35.3940	Hypothetical protein, conserved		N.D.
Lin17G12	2.49	1.3 ± 0.4	0.034	0	0	a	LinJ.19.0940	4-coumarate-CoA ligase		N.D.
Lin21H10	17.97	4.2 ± 1.0	0.019	0	0	b	LinJ.26.1670	Sphingolipid δ-4 desaturase, putative	+	4.1 ± 0.3
Lin22C9	2.14	1.1 ± 0.4	0.040	0	0	b	LinJ.33.2910	Ubiquitin-conjugating enzyme, putative		N.D.
27C6	4.17	2.1 ± 0.2	0.002	0	0	a	LinJ.31.1240	Vacuolar H^+^-translocating pyrophosphatase, putative		N.D.
Lin28C5	7.31	2.9 ± 0.6	0.020	0	0	b	LinJ.26.1670	Sphingolipid δ-4 desaturase, putative	+	4.1 ± 0.3
Lin31D11	3.00	1.6 ± 0.3	0.016	0	0	b	LinJ.31.1870	Protein kinase-like protein		N.D.
Lin34F1	2.98	1.6 ± 0.5	0.038				LinJ.08.1000	Histone deacetylase, putative		N.D.
							LinJ.26.1620	40S ribosomal protein S33, putative	+	92.2 ± 5.2
							LinJ.26.1630	40S ribosomal protein S33, putative	+	92.2 ± 5.2
							LinJ.26.1640	Hypothetical protein, conserved		N.D.
Lin41C12	2.95	1.6 ± 0.5	0.028	0	0	b	LinJ.31.1600	Cytochrome c oxidase VIII (coxVIII), putative		N.D.
Lin45A11	2.35	1.2 ± 0.4	0.033	0	0	b	LinJ.28.2220	Mitochondrial DEAD protein, putative		N.D.
Lin48B6	2.60	1.4 ± 0.4	0.026	0	0	b	LinJ.36.2050	Mismatch repair protein MSH8, putative		N.D.
Lin49B7	9.21	3.2 ± 1.1	0.039	0	0	b	LinJ.06.1320	Pteridin transporter, putative		N.D.
Lin50G5	3.83	1.9 ± 0.4	0.016	0	0	a	LinJ.21.2080	Cytochrome oxidase VI (coxVI), putative		N.D.
Lin51A8	2.24	1.2 ± 0.4	0.041	0	0	a	LinJ.32.4190	GIPL-galf transferase, putative		N.D.
Lin51E2	2.09	1.1 ± 0.2	0.018	0	0	b	LinJ.36.0020	Histone H4		N.D.
Lin51G7	3.85	1.9 ± 0.2	0.003	0	0	b	LinJ.19.1490	Oxidoreductase-like protein	+	3.6 ± 0.2
							LinJ.19.1500	Hypothetical protein, conserved		N.D.
Lin54C2	7.13	2.8 ± 0.5	0.010	0	0	a	LinJ.06.1310	Mitogen-activated protein kinase	+	10.8 ± 0.5
							LinJ.06.1320	Hypothetical protein, conserved		N.D.
							LinJ.06.1330	Hypothetical protein, conserved		N.D.
Lin58H6	5.43	2.4 ± 1.0	0.049	0	0	b	LinJ.08.0030	Vesicle-associated membrane protein, putative		N.D.
Lin60H10	4.41	2.1 ± 0.2	0.003	0	0	a	LinJ.23.0630	Oxidoreductase-like protein	+	2.9 ± 0.1
Lin76A1	3.08	1.6 ± 0.4	0.018	0	0	a	LinJ.31.3320	Histone H4	+	11.0 ± 0.4
Lin76F1	5.40	2.4 ± 0.5	0.013	0	0	b	LinJ.34.3370	Phosphatidylinositol 4-kinase, putative		N.D.
Lin77B12	2.01	1.0 ± 0.3	0.038	0	0	a	LinJ.27.1520	Eukaryotic translation initiation factor eIF4E, putative		N.D.
Lin80B3	2.83	1.5 ± 0.6	0.049	0	0	b	LinJ.28.3250	Glucose-6-phosphate N-acetyltransferase, putative		N.D.
Lin82D10	4.25	2.1 ± 0.5	0.019	0	0	a	LinJ.23.0040	β -propeller, putative	+	2.0 ± 0.1
							LinJ.23.0050	Peroxidoxin/Tryparedoxin peroxidase	+	21.2 ± 0.8
							LinJ.23.0060	Cyclophilin, putative		N.D.
Lin89D8	2.87	1.5 ± 0.4	0.023	0	0	a	LinJ.36.3230	Lipoate protein ligase, putative	+	8.0 ± 0.5
Lin93D6	5.50	2.5 ± 0.3	0.005	0	0	b	LinJ.26.1670/80	Sphingolipid δ-4 desaturase, putative	+	4.1 ± 0.4
							LinJ.26.1690	Cytochrome c oxidase, subunit V (coxV), putative		N.D.
Lin96H7	4.92	2.3 ± 0.3	0.007	0	0	a	LinJ.31.3310	Hypothetical protein, unknown function		N.D.
							LinJ.31.3320	Histone H4, putative	+	11.0 ± 0.4
Lin96B8	4.21	2.1 ± 0.8	0.046	0	0	a	LinJ.31.3310	Hypothetical protein, unknown function		N.D.
							LinJ.31.3320	Histone H4, putative	+	11.0 ± 0.4
Lin99G6	4.02	2.0 ± 0.5	0.002	4e-156	9e-80	b	LinJ.36.1730	Proteasome subunit β5, putative	+	5.2 ± 0.2
Lin105H8	3.92	2.0 ± 0.6	0.026	0	0	b	LinJ.36.3750	Cysteine synthase, putative	+	3.6 ± 0.0
Lin106G3	2.26	1.2 ± 0.3	0.029	0	0	a	LinJ.31.1070	Biotin/lipoate-protein ligase	+	8.0 ± 0.5
Lin110F5	3.55	1.8 ± 0.3	0.011	0	0	a	LinJ.16.1220	60S ribosomal protein L39, putative		N.D.
Lin111D8	9.80	3.3 ± 0.8	0.020	0	0	a	LinJ.08.1000	Histone deacetylase, putative		N.D.
Lin113B9	2.72	1.4 ± 0.6	0.048	0	0	a	LinJ.36.0550	Hypothetical protein, conserved		N.D.
							LinJ.36.0560	Protein phosphatase 2C, putative	+	6.4 ± 0.2
							LinJ.36.0570	Small nuclear ribonucleoprotein, putative	+	9.4 ± 0.8
Lin125F11	5.88	2.5 ± 1.0	0.046	7e-56	3e-55	a	LinJ.32.2780	Cistathionine γ-liase, putative		N.D.
Lin130C5	2.52	1.3 ± 0.5	0.040	3e-178	0	b	LinJ.36.3170	Exosome exonuclease RRP41, putative	-	-3.3 ± 0.2
							LinJ.36.3180	Clathrin coat assembly protein		N.D.
							LinJ.36.3190	Pre-mRNA branch site p14 protein, putative	+	43.2 ± 1.3
							LinJ.36.3200	Hypothetical protein, conserved		N.D.
Lin132A11	5.24	2.4 ± 0.1	0.001	0	0	a	LinJ.31.1240	Vacuolar H^+^-translocating pyrophosphatase, putative		N.D.
Lin136G4	2.66	1.4 ± 0.6	0.049	0	0	b	LinJ.22.1360	Hypothetical protein, conserved		N.D.
							LinJ.22.1370	60S ribosomal protein L14, putative	+	4.1 ± 0.3
Lin139D8	5.85	2.5 ± 0.6	0.003	0	0	b	LinJ.08.0010	Structural maintenance of chromosome protein 3, putative		N.D.
Lin146A12	2.67	1.4 ± 0.4	0.032	0	0	b	LinJ.30.0710	40S ribosomal protein S30, putative	+	56.2 ± 1.7
							LinJ.30.0720	NUDC-like protein		N.D.
Lin166F2	5.54	2.5 ± 0.7	0.023	0	0	b	LinJ.21.0770	Ribonuclease -L inhibitor, ABC subfamily E, putative		N.D.
Lin166H10	2.08	1.1 ± 0.2	0.014	0	0	b	LinJ.26.1680	Sphingolipid δ-4 desaturase, putative	+	4.1 ± 0.4
							LinJ.26.1690	Cytochrome b5 reductase, putative		N.D.
Lin168F2	2.17	1.1 ± 0.2	0.017	0	0	a	LinJ.32.0710	OSM-3-like kinesin		N.D.
Lin169E6	6.66	2.7 ± 0.9	0.037	0	0	b	LinJ.32.0550	Profilin, putative		N.D.
Lin172B9	4.42	2.1 ± 0.4	0.009	0	0	b	LinJ.26.1680	Sphingolipid δ-4 desaturase, putative	+	4.1 ± 0.4
							LinJ.26.1690	Cytochrome b5 reductase, putative		N.D.
Lin208F7	3.33	1.7 ± 0.6	0.033	0	0	b	LinJ.30.3640	Ser/Thr protein kinase, putative		N.D.
Lin276F6	3.47	1.8 ± 0.3	0.007	0	0	b	LinJ.35.2370	Protein kinase, putative		N.D.
Lin290F2	3.52	1.8 ± 0.3	0.012	0	0	b	LinJ.04.1250	Actin		N.D.
Lin298H2	7.23	2.8 ± 0.5	0.011	0	0	b	LinJ.22.1340	Ser/Thr protein phosphatase		N.D.
Lin299A1	4.34	2.1 ± 0.8	0.045	0	0	b	LinJ.36.1720	Universal minicircle sequence binding protein (UMSBP), putative		N.D.
Lin18A12	-2.20	-1.1 ± 0.4	0.044	0	0	b	LinJ.33.2430	UDP-glucose 4′-epimerase		N.D.
Lin25B7	-2.34	-1.2 ± 0.4	0.034	0	0	b	LinJ.31.3390	Sodium stibogluconate resistance protein		N.D.
Lin30H4	-3.45	-1.8 ± 0.4	0.017	0	2e-111	b	LinJ.27.2500	Glycosomal phosphoenolpyruvate carboxykinase, putative		N.D.
Lin35H4	-2.77	-1.5 ± 0.5	0.039	0	0	b	LinJ.34.3740	Expression-site associated glycoprotein (ESAG5), putative		N.D.
Lin49D6	-2.83	-1.5 ± 0.5	0.031	0	1e-152	b	LinJ.19.0590	Protein kinase, putative		N.D.
Lin54A3	-2.21	-1.1 ± 0.4	0.037	4e-156	0	b	LinJ.36.6510	Small G protein, putative		N.D.
Lin77H8	-2.07	-1.0 ± 0.4	0.039	0	0	b	LinJ.08.0690	Amastin-like protein		N.D.
Lin88B2	-2.08	-1.1 ± 0.4	0.040	0	0	b	LinJ.10.1070	Histone H3		N.D.
Lin101D5	-2.54	-1.3 ± 0.3	0.017	0	2e-28	b	LinJ.27.2500	Glycosomal phosphoenolpyruvate carboxykinase, putative		N.D.
Lin101E5	-2.71	-1.4 ± 0.5	0.046	0	0	b	LinJ.35.5330	Protein kinase, putative		N.D.
Lin107B10	-2.22	-1.1 ± 0.3	0.003	0	0	b	LinJ.06.1110	Deoxyribose phosphate aldolase, putative	+	-7.3 ± 0.6
							LinJ.06.1120	Hypothetical protein, conserved		N.D.
Lin115H5	-2.37	-1.2 ± 0.4	0.034	0	3e-136	b	LinJ.03.0790	6-phosphofructo-2-kinase/fructose-2,6-diphosphatase, putative		N.D.
Lin123E6	-2.11	-1.1 ± 0.3	0.019	0	0	b	LinJ.23.0980	Actin-interacting protein		N.D.
Lin188B12	-2.46	-1.3 ± 0.1	0.001	0	0	b	LinJ.31.3400	Sodium stibogluconate-resistance protein		N.D.
Lin286D1	-2.41	-1.3 ± 0.4	0.035	0	0	b	LinJ.08.1320	Amastin-like protein		N.D.
Lin274G6	-2.25	-1.2 ± 0.4	0.037	0	0	b	LinJ.08.0680/90	Amastin-like protein		N.D.
Lin283F3	-2.12	-1.1 ± 0.3	0.023	0	0	b	LinJ.15.0130	ATP-dependent RNA helicase, putative		N.D.
Lin283H1	-2.60	-1.4 ± 0.2	0.010	0	0	b	LinJ.21.1670	2-oxoisovalerate dehydrogenase, subunit α, putative		N.D.
Lin308D6	-2.11	-1.1 ± 0.1	0.002	0	0	b	LinJ.11.0060	Protein kinase, putative		N.D.

Fold changes (up-regulation if F > 2, over the dividing line, and down-regulation if F < -2, below the dividing line), base-two logarithmic scale F and their SD, p, e-values, clone definitions according to mapping outcomes a, b and c, Ids. and annotated functions in the *L. infantum* genome sequence and the qRT-PCR outcomes are provided. When a given clone overlaps with more than one annotation, only the clones checked for differential regulation using qRT-PCR and non-resolved clones overlapping with resolved clones containing a common gene appear in this table.

### qRT-PCR validation

This approach has been useful not only for the validation of microarray results, but also to sort out the differentially regulated genes in clones fulfilling the cutoff values in the microarray hybridization analysis that align with more than one gene. All these data are reflected in Table 2 and according to them, five genes of known function already resolved by microarrays themselves have been confirmed by qRT-PCR and 16 clones not directly resolved by microarray analysis contain at least one differentially regulated gene. Constant expression values for a given CDS have been obtained only in clones that overlap with more than one CDS. The remaining gene is presumed to be differentially regulated except if more than two CDS overlap with the clone. Consequently, 7.8% of differentially regulated genes have been validated and we have not detected any differing result between the techniques so far, including those in previous studies [16, 23–26].

### Differential gene expression between Pro-Pper and amastigotes

**Figure 3:**
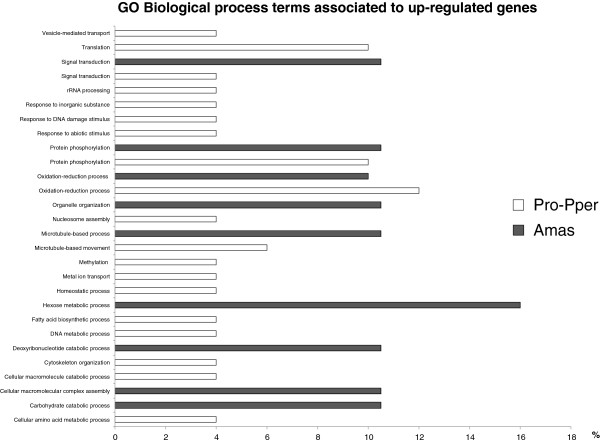
**Biological process multi-level bar graph for GO terms annotated with BLAST2GO.**

**Figure 4:**
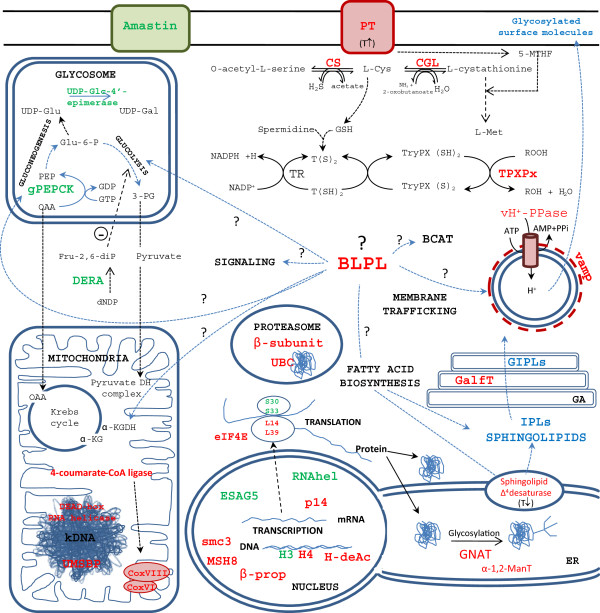
**Schema illustrating the scenario of the relative expression profiles of Pro-Pper and amastigotes.** Protein products of regulated genes in Pro-Pper/A are represented in red and those of down-regulated genes in green. Blue arrows highlight the hypothesis for an important role for the BLPL, which may be specifically regulated to achieve any of the processes indicated. Differentially regulated genes related to signal transduction: calmodulin-like EF-hand protein, MAPKs, PI4K, PKs, PP2C, Ser/Thr PPase, small G protein. Differentially regulated genes related to cytoskeleton remodeling: actin microfilament, AIP, coronin, α-tubulin isoform.

The differences found in molecular functions and biological processes are summarized along with the outcome of the BLAST2GO analysis (Figure 3) and the schema based on information from the described analysis, including the cell component terms, literature and GeneDB, TriTrypDB and KEGG [39] databases (Figure 4). Processes related to DNA metabolism, chromosome organization, translation, cellular response to stimulus and stress, transport and movement are associated with up-regulated genes in Pro-Pper with respect to amastigotes (Figure 3). Overall, these data suggest a more active metabolic status of promastigotes, which is in agreement with previously reported data [23]. Table 2 contains stage-specific regulated genes of known function and the differentially regulated hypothetical protein genes are included in the Additional file 4. Regarding this transcriptome variation (Figure 4), significant changes in metabolism may take place between promastigotes from the anterior gut of *P. perniciosus* and intracellular amastigotes. The biotin/lipoate ligase genes LinJ.31.1070 and LinJ.36.3230 (BLPL) are over-expressed in Pro-Pper, which suggests an increased demand for lipoic acid and/or biotin by any dehydrogenase complex and/or carboxylase, respectively. In fact, the genes encoding BLPLs bear the activity EC 6.3.4.15 and the activities 6.3.4.9., 6.3.4.10 and 6.3.4.11 are absent in *L. infantum* (KEGG database). This suggests an important biological role of BLPLs in these parasites. One hypothesis for the central role of this protein in the Pro-Pper/A scenario is highlighted in Figure 4. One of the enzymes demanding the cofactor could be the glycosomal phosphoenolpyruvate carboxykinase (gPEPCK). Provided that the level of gPEPCK transcripts are higher in amastigotes, the expression profile of BLPL may not specially favor gluconeogenesis in Pro-Pper. *L. donovani* amastigotes also over-express gPEPCK with respect to cultured promastigotes [29]. Another possibility may be increased activity of carboxylases participating in leucine and isoleucine degradation in Pro-Pper but again this is not likely to occur provided up-regulation of the α-ketoisovalerate dehydrogenase gene (KIVDH) in amastigotes. This gene was also found to be up-regulated at the protein level in mature *L. donovani* amastigotes [29]. As a consequence, the up-regulation of this gene in amastigotes takes place independently of the source of promastigotes (culture or foregut of the sand fly). BLPL is not only essential for the branched-chain oxoacid dehydrogenase complex but also for the pyruvate dehydrogenase and the α-ketoglutarate dehydrogenase complexes. As a difference with some genes involved in electron transport chain, none of the genes encoding proteins involved in pyruvate decarboxylation and the Krebs cycle are differentially regulated between Pro-Pper and amastigotes. The expression profile of BLPL may also be associated with fatty acid biosynthesis by the acetyl-CoA carboxylase. Up-regulation of the sphingolipid-Δ^4^-desaturase cluster and the glycosylinositol phospholipid:galactofuranose (GIPL-galf) transferase gene in Pro-Pper (Table 2) suggests a possible increase of the demand of fatty acids in Pro-Pper. In fact, large amounts of unglycosylated inositolphosphoceramide molecules (IPC) [42] and GIPLs appear on the surface of the parasite and fatty acids are required for the biosynthesis of the corresponding lipid anchor. Palmitic acid is required for sphingosine biosynthesis, whereas the function of the GIPL-galf transferase is to add a galactofuranose residue to the exposed end of the molecule in GIPL-1 and close to the end in others, once the phospholipid anchor has been synthesized and fatty acids modified [43]. Sphingosine, ceramide and their phosphorylated derivatives are also signaling molecules as well as phospholipids, such as phosphatidic acid, *lyso*-phospholipids and phosphatidylinositol (PI). These molecules also participate in membrane trafficking and cytoskeleton remodeling. These facts also suggest an indirect role of BLPL and sphingolipid-Δ^4^-desaturase gene up-regulation in signaling in Pro-Pper, which is in agreement with the up-regulation of phosphatidylinositol 4-kinase (PI4K) (Table 2). However, these processes are activated by small G proteins at least in other eukaryotes [42] and the expression levels of the only annotated gene that encodes for this type of proteins in the *L. infantum* genome is over-expressed in amastigotes. As signaling pathways have not been yet elucidated in *Leishmania* spp. it is important to note that a correspondence in these processes between other eukaryotes, such as yeasts and mammals and the parasite may not be certain. Regarding vacuoles, genes encoding a vacuolar-associated membrane protein and a vacuolar proton translocating pyrophosphatase are up-regulated in Pro-Pper (Table 2), which may be related to an indirect membrane trafficking triggered by the up-regulation of sphingolipid-Δ4-desaturase and PI4K.

The up-regulation of the cysteine synthase (CS) and the cystathionine γ-lyase (CGL) genes in Pro-Pper (Table 2) suggests an increase of L-cysteine and, most likely, L-methionine biosynthesis. In addition, glutathione is synthesized from L-cysteine. This may be related to the over-expression of tryparedoxin peroxidase (TPXPx) that has been detected in Pro-Pper (Figure 4, Table 2). The pteridine transporter LinJ.06.1320 (PT) is also up-regulated at this stage compared to amastigotes. This difference was also found between cultured promastigotes and amastigote-like forms obtained by increasing the temperature from 27 to 37°C with and without a simultaneous pH decrease to 4.5. Thus, a temperature increase is responsible for the down-regulation of this gene [16]. The same expression profile of PT was found in *L. mexicana* [21], *L. major* [27] and *L. infantum* [16] amastigote-like forms and also *L. infantum* intracellular amastigotes [23]. Pterins are required for the biosynthesis of several amino acids such as methionine. This is most likely related to the up-regulation of CS and CGL, although an opposite expression pattern was found for the LinJ.10.0410 and LinJ.14.1440 genes, which encode PT isoforms in amastigote-like forms and amastigotes [16, 23]. Nevertheless, these differences have not been found between Pro-Pper and amastigotes. Taken together, these data suggest that only PT LinJ.06.1320 is actually up-regulated in amastigotes in the natural life cycle of the parasite due to temperature increase and the other differences in transcript abundance may be related to the use of the culture model.

The gPEPCK expression profile may be also affected by serum in the culture medium. In fact, it is down-regulated under serum depletion (unpublished result), over-expressed in stationary compared to logarithmic phase promastigotes [23] and up-regulated in amastigotes with respect to Pro-Pper, but is not differentially expressed between amastigotes and cultured promastigotes. The inhibition of glycolysis in amastigotes may be carried out by fructose-2,6-diphosphate, as the 6-phosphofructo-2-kinase gene is up-regulated at this stage. Consequently, the role of gPEPCK up-regulation for monosaccharide supplies may be to accomplish the biosynthesis of glycoconjugates and/or sugar-derived metabolites. These findings are in agreement with the absence of monosaccharide sources in the environment of amastigotes, which has been previously reported [44]. In fact, it has been reported that promastigotes and amastigotes of *Leishmania* spp. can use amino acids as their major or only carbon source [45]. The up-regulation of the deoxyribose phosphate aldolase EC 4.1.2.4 (DERA) gene, which is involved in deoxyribose phosphate catabolic processes (GO0046386), suggests that another possible source for amastigotes could be deoxynucleotide degradation, which may be taken from the environment. The products of the reaction catalyzed by DERA (acetaldehyde and glyceraldehydes-3-phosphate) are used as energy and carbon sources.

The glucose-6-phosphate N-acetyltransferase gene (GNAT) is down-regulated in amastigotes not only with respect to cultured logarithmic and stationary phase promastigotes [23] but also Pro-Pper. Although acidification alone leads to an increase of GNAT transcript abundance, the down-regulation of GNAT in amastigotes is due to the combined effect of temperature increase and pH decrease, as acidification does not lead to differentiation into amastigotes itself [16]. According to these data, GNAT transcript levels are less abundant in amastigotes than in promastigotes regardless of their origin (culture or foregut of the sand fly).

The following genes involved in gene expression regulation and intracellular signalling are up-regulated in Pro-Pper: eukaryotic initiation factor 4E (eIF4E), three ribosomal proteins (L39, S30 and S33), a small nuclear ribonucleoprotein, the pre-mRNA branch site p14 protein, a MAP kinase, a protein phosphatase 2C (PP2C), a Ser/Thr protein phosphatase, three protein kinases, a β-propeller protein, an EF hand-containing calmodulin-like protein, sphingolipid-Δ^4^-desaturase and the PI4K. Only the expression site-associated glycoprotein 5 (ESAG5) and an ATP-dependent DNA helicase genes are up-regulated in amastigotes. These findings suggest that a decrease in gene expression regulation and signaling activities take place in the differentiation process of promastigotes to amastigotes, which is in agreement with the lower up-regulation rate observed in amastigotes with respect to promastigotes independently of their origin (culture or foregut of the sand fly) (see [23] for cultured promastigotes and the next subsection for uncultured promastigotes). The pre-mRNA branch site p14 protein gene is also over-expressed in response to pH increase itself, providing additional evidence that this factor has limited influence on the differentiation process to amastigotes [16]. The amastin genes of cluster LinJ08.0680/0690/720/1320 are up-regulated in *L. infantum* amastigotes with respect to both cultured promastigotes [15, 16] and Pro-Pper (Table 2). Using the axenic culture model, it was found that the temperature increase, which is essential for differentiation of promastigotes to amastigotes, triggers the up-regulation of these genes [16]. These coincidences are not applicable for the surface sodium stibogluconate resistance protein (SbGRP) gene, as it shows the opposite expression profile between cultured promastigotes [23] and Pro-Pper (Table 2) with respect to amastigotes, a difference that may be due to the different environmental conditions inside the gut of the sand fly and axenic cultures. As for the microtubule cytoskeleton, this type of observations has also been made for the expression profile of the α-tubulin LinJ.13.1450, which is up-regulated in amastigotes with respect to Pro-Pper but was previously found to be down-regulated in amastigotes compared to *L. donovani* stationary phase promastigotes [29] and *L. infantum* stationary phase promastigotes [23]. Additionally, the OSM3 kinesin gene is up-regulated in Pro-Pper instead. The reason for the over-expression of the α-tubulin gene in amastigotes is unknown as is a similar difference in the microfilament cytoskeleton, namely in an actin-interacting protein. Further investigations of these changes may reveal whether they are involved in the morphological changes these parasites undergo.

### More clues about promastigote pre-adaptation

We described that the over-expression rate (number of up-regulated genes in a given stage or condition compared to the other one) is reduced in amastigotes with respect to cultured promastigotes of different *Leishmania* spp. [23], which supports the hypothesis of pre-adaptation of promastigotes, as stated by several authors [46, 47]. In this case, the term pre-adaptation is understood to be the preparation in advance for intracellular survival once infection and differentiation to amastigotes occur. In fact, it has been reported that in some cases, amastigote-like forms are found within the population of metacyclic promastigotes located in the gut section anterior to the stomodeal valve of *P. papatasi* infected with *L. major*, which is most likely induced by respective slight temperature increase and a pH decrease after the female sand fly feeds [48]. This is in agreement with our previous findings about the effects of temperature and pH in the transcriptome during differentiation in *L. infantum* [16]. Tang et al. [49] measured the pH of the thoracic and abdominal mid gut of the sand fly *Lutzomyia longipalpis* concluding that before blood feeding, the pH is neutral in the thoracic mid gut and is alkaline in the abdominal mid gut and thereafter it diminishes to 6.8 or less. The pH in the parasitophorous vacuole of phagocytes of the mammalian host is between 4.5 and 5.5 and the temperature is about 37°C in the case of species responsible for visceral leishmaniasis. These findings support the hypothesis of pre-adaptation of promastigotes towards differentiation to amastigotes that has been previously proposed [46, 47].

A binomial test has been performed for the set of differentially regulated genes between amastigotes and Pro-Pper (absolute frequencies in Table 1), and the outcome confirms a decrease of up-regulated genes in amastigotes with respect to Pro-Pper (p < 0.0001), as it was reported using the culture model [23]. Overall, gene expression, signaling and response to stimulus, movement and response to stress are processes associated with up-regulated genes in Pro-Pper (Figure 3), suggesting a more active general metabolic status of promastigotes than amastigotes, which is consistent with the lower up-regulation rate in amastigotes and constitutes an additional evidence of the preadaptation hypothesis. Cellular component GO terms are in agreement, as ribosomes, the nucleolus, the nucleosome, the cytoskeleton and the proteasome are locations associated to some of the over-expressed genes. The genes involved in regulation of gene expression and intracellular signaling may be of special relevance. Thus, the expression profile of the biosynthetic gene sphingolipid-Δ^4^-desaturase gene may suggest an important role for IPC molecules in the differentiation of promastigotes to amastigotes, as they are involved in some of these important processes.

The GIPL-galf transferase is another gene up-regulated in Pro-Pper that may have an important role in pre-adaptation. In fact, McConville et al. [50] proposed that the GIPL molecules are present in all stages but are more abundant in amastigotes due to the relative decrease of glycoproteins, lipophosphoglycan (LPG) and proteophosphoglycan (PPG) on the amastigote surface and that the GIPLs protect other proteins of the plasma membrane against the lytic enzymes of the parasitophorous vacuole. Moreover, GIPL-1 plays a role in the interaction of promastigotes and amastigotes with macrophages [51]. The terminal galactofuranose residue it contains may be involved in macrophage recognition through a putative receptor that has been previously reported [51]. Therapeutic targets of the galactofuranose biosynthetic pathway have also been recently described in kinetoplastids causing leishmaniasis and Chagas disease [52]. As mentioned before, the up-regulation of the biotin/lipoate ligase gene may be indirectly linked to the increase of GIPL biosynthesis in Pro-Pper.

The expression pattern of some of the genes of the amastin superfamily also provides a clue. Cultured stationary phase promastigotes show over-expression of amastin genes when compared to logarithmic phase amastigotes [23] but the highest levels are reached in amastigotes and cultured amastigote-like forms compared to cultured promastigotes [15, 16] or Pro-Pper. Although their role is unknown, these glycoproteins of the surface of amastigotes seem to be important for pathogenesis. In fact, Bolhassani et al. [53] reported partial protection in mice conferred by the amastin sequence fused to the VSP22 protein of herpes simplex virus 1 administered as a DNA vaccine.

### The influence of the promastigote culture model in stage-specific gene regulation

**Figure 5:**
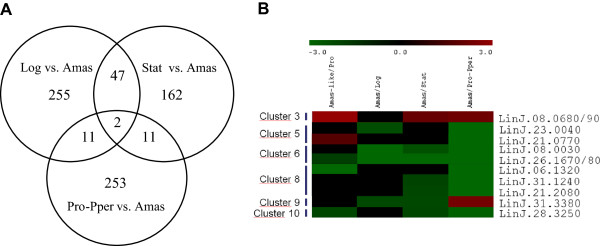
**Comparison of the whole genome gene expression profile of amastigotes using Pro-Pper or cultured promastigotes as reference.** The profiles of cultured logarithmic and stationary phase promastigotes compared with amastigotes have been published [23]. **(A)** Venn diagrams contrasting differential gene expression in *L. infantum* amastigotes depending on the origin of promastigotes. **(B)** Relative expression and classification (MEV analysis) in clusters of genes showing similar patterns or opposite expression profiles in amastigotes depending on the source of promastigotes (see a complete overview of the clustering analysis in Additional file 5).

The differential gene expression profiles between *L. infantum* promastigotes and amastigotes have been studied using cultured promastigotes either in logarithmic or stationary phase [23] and promastigotes isolated from the anterior gut of the sand fly *P. perniciosus* (this work). The comparison between these analyses using promastigotes from culture and from the sand fly has been performed by Venn diagram and iterative hierarchical clustering (Figure 5, Additional file 5) and suggests that the culture conditions affect certain aspects of differentiation of promastigotes to amastigotes related to differential transcript abundance. In fact, only two genes (vesicle-associated membrane protein and sphingolipid-Δ4-desaturase, both down-regulated in amastigotes) show the same expression pattern between Pro-Pper and cultured logarithmic and stationary phase promastigotes. The number of similarities in the stage-specific expression profile during differentiation to amastigote between logarithmic and Pro-Pper is 11 genes, as well as for stationary phase vs. Pro-Pper. Some of these genes have known function: the histone H4 LinJ.36.0020 gene, the β-propeller protein LinJ.23.0040 and the histone deacetylase LinJ.08.1000, which differed between logarithmic phase promastigotes and Pro-Pper (all up-regulated with regard to amastigotes) and coxVI, GNAT, amastins of the LinJ.08.0680 cluster and the vacuolar proton-translocating pyrophosphatase, which differed between stationary phase promastigotes and Pro-Pper (all up-regulated with respect to amastigotes except the amastins). In addition, the profile of SbGRP and the α-tubulin LinJ13.1450 is opposite between promastigotes in culture and in the anterior gut of the sand fly with respect to amastigotes. There are several coincidences with the outcome of the high-throughput iTRAQ-based proteome analysis described by Rosenzweig et al. [29], as 2-oxoisovalerate dehydrogenase, DERA and gPEPCK are up-regulated in *L. donovani* amastigotes vs. axenically cultured promastigotes, as well as in *L. infantum* vs. Pro-Pper, whereas the ATP-dependent RNA helicase LinJ.15.0130 shows an opposite pattern.

An illustrative example in the transcriptome profiles of sand fly-derived and cultured promastigotes during differentiation towards the amastigote stage is the up-regulation of the pre-mRNA branch site protein p14 gene in Pro-Pper with respect to amastigotes considered together with down-regulation after the treatment of promastigotes with pH 4.5 [16]. pH is more acidic in a promastigote stationary culture (5.5-6.0) than in the thoracic mid gut and likely in the stomodeal valve (6.8 or lower according to Tang et al. [49]) and amastigotes are capable of withstanding pH values between 4.5 and 5.5. Thus, acidification turns the over-expression of the p14 gene in the slightly acidic environment of the *P. perniciosus* anterior gut into constant expression in more acidified stationary phase cultures and a further decrease of pH of the culture medium leads to under expression of this gene in forms of the parasite with differentially expressed transcriptome quite distinct from the natural promastigote and amastigote stages [16].

## Conclusions

The differential expression profile of promastigotes to amastigotes, considering the initial and final time points (metacyclic promastigotes and amastigotes), is notably different when the source of metacyclic promastigotes is the foregut of the sand fly instead of axenic cultures. This finding suggests that using promastigote cultures may affect certain aspects of studying the parasite.

### Availability of the supporting data

All data concerning the shotgun genomic DNA microarrays used and the hybridization procedure have been deposited in the GEO repository complying MIAME (http://www.ncbi.nlm.nih.gov/geo/query/acc.cgi?acc=GSE11269). Particular information about the sequences of primers and TaqMan probes used, hybridization controls in the microarray experiment, hypothetical proteins and analysis by gene clustering is available in the additional files that have been submitted along with this manuscript.

## Electronic supplementary material

Additional file 1: **Primers and TaqMan-MGB probes used for qRT-PCR validation and the determination of differential expression in unresolved clones. Table S1.** Sequences of qRT-PCR primers and probes. (PDF 212 KB)

Additional file 2: **Clones that map with UTRs or less than 5% of length of an ORF.** **Table S2.** Clones that do not fulfill the criteria specified in section the Methods section, Microarray hybridization and analysis of data subsection. (DOC 205 KB)

Additional file 3: **Microarray controls.** **Table S3.** The results of the Pro-Per/A cDNA-microarray hybridization analysis for positive and negative control spots. (DOCX 18 KB)

Additional file 4: **Hypothetical proteins.** **Table S4.** Hypothetical proteins up-regulated in Pro-Pper/A. **Table S5.** Hypothetical proteins down-regulated in Pro-Pper/A. (DOC 168 KB)

Additional file 5: **Overview of the MEV clustering analysis.** **Figure S1.** Profile of clusters of genes differentially regulated in amastigotes. (PPTX 310 KB)

## Abbreviations

ABCE: ATP-binding cassette subfamily E
BLPL: Biotin/lipoate protein ligase
CGL: Cystathionine γ-lyase
cox: Cytochrome c oxidase
4CL: 4-coumarate-CoA ligase
CS: Cysteine synthase
DERA: Deoxyribose phosphate aldolase
eIF4E: Eukaryotic initiation factor 4E
ESAG5: Expressed-site associated glycoprotein 5
GIPL: Glycosylinositolphospholipid
GIPL-galfT: glycosylinositolphospholipid:galactofuranose transferase
GNAT: Glucose-6-phosphate N-acetyltransferase
gPEPCK: Glycosomal phosphoenolpyruvate carboxykinase
H-deAc: Histone deacetylase
HIFBS: Heat inactivated foetal bovine serum
IPL: Inositolphospholipid
IPC: Inositolphosphoceramide
α-KGDH: α-ketoglutarate dehydrogenase
KIVDH: α-ketoisovalerate dehydrogenase
LPG: Lipophosphoglycan
α-1: 2-ManT: α-1,2-mannosyltransferase
MSH8: Mismatch repair protein 8
5-MTHF: 5-methyltetrahydrofolate
OAA: Oxalacetate
NFQ: Non-fluorescent quencher
β-prop: β-propeller protein
PI: Phosphatidylinositol
PI4K: Phosphatidylinositol 4-kinase
PK: Protein kinase
PP2C: Protein phosphatase 2C
PPG: Proteophosphoglycan
PT: Pteridine transporter
qRT-PCR: Real time quantitative RT-PCR
sn-RNP: small nuclear ribonucleoprotein
SbGRP: Sodium stibogluconate resistance protein
TPXPx: Tryparedoxin peroxidase
TR: Trypanothione reductase
TryPX[SH]_2_: Reduced tryparedoxin
TryPX[S]_2_: Oxidized tryparedoxin
T[SH]_2_: Reduced trypanothione
T[S]_2_: Oxidized trypanothione
vamp: Vesicle-associated membrane protein
vH^+^-PPase: Vacuolar proton translocating pyrophosphatase
UBC: Ubiquitin-conjugating enzyme.

## References

[1] Desjeux P (1996). Leishmaniasis. Public health aspects and control. Clin Dermatol.

[2] WHO (2010). Report of a Meeting of the WHO Expert Committee on the Control of Leishmaniases.

[3] Pasquau F, Ena J, Sanchez R, Cuadrado JM, Amador C, Flores J, Benito C, Redondo C, Lacruz J, Abril V, Onofre J (2005). Leishmaniasis as an opportunistic infection in HIV-infected patients: determinants of relapse and mortality in a collaborative study of 228 episodes in a Mediterreanean region. Eur J Clin Microbiol Infect Dis.

[4] Cruz I, Nieto J, Moreno J, Canavate C, Desjeux P, Alvar J (2006). Leishmania/HIV co-infections in the second decade. Indian J Med Res.

[5] Arce A, Estirado A, Ordobas M, Sevilla S, Garcia N, Moratilla L, de la Fuente S, Martinez AM, Perez AM, Aranguez E, Iriso A, Sevillano O, Bernal J, Vilas F (2013). Re-emergence of leishmaniasis in Spain: community outbreak in Madrid, Spain, 2009 to 2012. Euro Surveill.

[6] Molina R, Jimenez MI, Cruz I, Iriso A, Martin-Martin I, Sevillano O, Melero S, Bernal J (2012). The hare (Lepus granatensis) as potential sylvatic reservoir of Leishmania infantum in Spain. Vet Parasitol.

[7] Lucientes-Curdi J, Benito-de-Martin MI, Castillo-Hernandez JA, Orcajo-Teresa J (1991). Seasonal dynamics of Larroussius species in Aragon (N.E. Spain). Parassitologia.

[8] Killick-Kendrick R (1999). The biology and control of phlebotomine sand flies. Clin Dermatol.

[9] Neal RA, Miles RA (1963). Heated blood agar medium for the growth of Trypanosoma cruzi and some species of Leishmania. Nature.

[10] Lemma A, Schiller EL (1964). Extracellular cultivation of the leishmanial bodies of species belonging to the protozoan genus leishmania. Exp Parasitol.

[11] Steiger RF, Steiger E (1976). A defined medium for cultivating Leishmania donovani and L. braziliensis. J Parasitol.

[12] Berens RL, Marr JJ (1978). An easily prepared defined medium for cultivation of Leishmania donovani promastigotes. J Parasitol.

[13] Zilberstein D, Myler P, Fassel N (2008). Physiological and Biochemical Aspects of Leishmania Development. Leishmania After the Genome.

[14] Zuckerman A, Lainson R, Kreier JP (1977). Leishmania. Parasitic Protozoa.

[15] Rochette A, Raymond F, Corbeil J, Ouellette M, Papadopoulou B (2009). Whole-genome comparative RNA expression profiling of axenic and intracellular amastigote forms of Leishmania infantum. Mol Biochem Parasitol.

[16] Alcolea PJ, Alonso A, Gomez MJ, Sanchez-Gorostiaga A, Moreno-Paz M, Gonzalez-Pastor JE, Toraño A, Parro V, Larraga V (2010). Temperature increase prevails over acidification in the gene expression modulation of amastigote differentiation in Leishmania infantum. BMC Genomics.

[17] Akopyants NS, Matlib RS, Bukanova EN, Smeds MR, Brownstein BH, Stormo GD, Beverley SM (2004). Expression profiling using random genomic DNA microarrays identifies differentially expressed genes associated with three major developmental stages of the protozoan parasite Leishmania major. Mol Biochem Parasitol.

[18] Almeida R, Gilmartin BJ, McCann SH, Norrish A, Ivens AC, Lawson D, Levick MP, Smith DF, Dyall SD, Vetrie D, Freeman TC, Coulson RM, Sampaio I, Schneider H, Blackwell JM (2004). Expression profiling of the Leishmania life cycle: cDNA arrays identify developmentally regulated genes present but not annotated in the genome. Mol Biochem Parasitol.

[19] Saxena A, Lahav T, Holland N, Aggarwal G, Anupama A, Huang Y, Volpin H, Myler PJ, Zilberstein D (2007). Analysis of the Leishmania donovani transcriptome reveals an ordered progression of transient and permanent changes in gene expression during differentiation. Mol Biochem Parasitol.

[20] Saxena A, Worthey EA, Yan S, Leland A, Stuart KD, Myler PJ (2003). Evaluation of differential gene expression in Leishmania major Friedlin procyclics and metacyclics using DNA microarray analysis. Mol Biochem Parasitol.

[21] Holzer TR, McMaster WR, Forney JD (2006). Expression profiling by whole-genome interspecies microarray hybridization reveals differential gene expression in procyclic promastigotes, lesion-derived amastigotes, and axenic amastigotes in Leishmania mexicana. Mol Biochem Parasitol.

[22] Lahav T, Sivam D, Volpin H, Ronen M, Tsigankov P, Green A, Holland N, Kuzyk M, Borchers C, Zilberstein D, Myler PJ (2011). Multiple levels of gene regulation mediate differentiation of the intracellular pathogen Leishmania. FASEB J.

[23] Alcolea PJ, Alonso A, Gomez MJ, Moreno I, Dominguez M, Parro V, Larraga V (2010). Transcriptomics throughout the life cycle of Leishmania infantum: high down-regulation rate in the amastigote stage. Int J Parasitol.

[24] Alcolea PJ, Alonso A, Larraga V (2011). Genome-wide gene expression profile induced by exposure to cadmium acetate in Leishmania infantum promastigotes. Int Microbiol.

[25] Alcolea PJ, Alonso A, Larraga V (2011). Proteome profiling of Leishmania infantum promastigotes. J Eukaryot Microbiol.

[26] Alcolea PJ, Alonso A, Sanchez-Gorostiaga A, Moreno-Paz M, Gomez MJ, Ramos I, Parro V, Larraga V (2009). Genome-wide analysis reveals increased levels of transcripts related with infectivity in peanut lectin non-agglutinated promastigotes of Leishmania infantum. Genomics.

[27] Leifso K, Cohen-Freue G, Dogra N, Murray A, McMaster WR (2007). Genomic and proteomic expression analysis of Leishmania promastigote and amastigote life stages: the Leishmania genome is constitutively expressed. Mol Biochem Parasitol.

[28] Rochette A, Raymond F, Ubeda JM, Smith M, Messier N, Boisvert S, Rigault P, Corbeil J, Ouellette M, Papadopoulou B (2008). Genome-wide gene expression profiling analysis of Leishmania major and Leishmania infantum developmental stages reveals substantial differences between the two species. BMC Genomics.

[29] Rosenzweig D, Smith D, Opperdoes F, Stern S, Olafson RW, Zilberstein D (2008). Retooling Leishmania metabolism: from sand fly gut to human macrophage. Faseb J.

[30] Sundstrom C, Nilsson K (1976). Establishment and characterization of a human histiocytic lymphoma cell line (U-937). Int J Cancer.

[31] Minta JO, Pambrun L (1985). In vitro induction of cytologic and functional differentiation of the immature human monocytelike cell line U-937 with phorbol myristate acetate. Am J Pathol.

[32] Hart DT, Vickerman K, Coombs GH (1981). A quick, simple method for purifying Leishmania mexicana amastigotes in large numbers. Parasitology.

[33] Molina R (1991). Laboratory adaptation of an autochtonous colony of Phlebotomus perniciosus Newstead, 1911 (Diptera: Psychodidae). Res Rev Parasitol.

[34] Jimenez M, Gonzalez E, Iriso A, Marco E, Alegret A, Fuster F, Molina R (2013). Detection of Leishmania infantum and identification of blood meals in Phlebotomus perniciosus from a focus of human leishmaniasis in Madrid. Spain Parasitol Res.

[35] Rastrojo A, Carrasco-Ramiro F, Martin D, Crespillo A, Reguera RM, Aguado B, Requena JM (2013). The transcriptome of Leishmania major in the axenic promastigote stage: transcript annotation and relative expression levels by RNA-seq. BMC Genomics.

[36] Conesa A, Gotz S, Garcia-Gomez JM, Terol J, Talon M, Robles M (2005). Blast2GO: a universal tool for annotation, visualization and analysis in functional genomics research. Bioinformatics.

[37] **GeneDB** http://www.genedb.org/Homepage/Linfantum

[38] **TriTrypDB** http://tritrypdb.org/tritrypdb/

[39] **KEGG: Kyoto Encyclopedia of Genes and Genomes** http://www.genome.jp/kegg/

[40] Ivens AC, Peacock CS, Worthey EA, Murphy L, Aggarwal G, Berriman M, Sisk E, Rajandream MA, Adlem E, Aert R, Anupama A, Apostolou Z, Attipoe P, Bason N, Bauser C, Beck A, Beverley SM, Bianchettin G, Borzym K, Bothe G, Bruschi CV, Collins M, Cadag E, Ciarloni L, Clayton C, Coulson RM, Cronin A, Cruz AK, Davies RM, De Gaudenzi J (2005). The genome of the kinetoplastid parasite, Leishmania major. Science.

[41] Peacock CS, Seeger K, Harris D, Murphy L, Ruiz JC, Quail MA, Peters N, Adlem E, Tivey A, Aslett M, Kerhornou A, Ivens A, Fraser A, Rajandream MA, Carver T, Norbertczak H, Chillingworth T, Hance Z, Jagels K, Moule S, Ormond D, Rutter S, Squares R, Whitehead S, Rabbinowitsch E, Arrowsmith C, White B, Thurston S, Bringaud F, Baldauf SL (2007). Comparative genomic analysis of three Leishmania species that cause diverse human disease. Nat Genet.

[42] Zhang K, Barron T, Turco SJ, Beverley SM (2004). The LPG1 gene family of Leishmania major. Mol Biochem Parasitol.

[43] McConville MJ, Ferguson MAJ (1993). The structure, biosynthesis and function of glycosylated phosphatidylinositols in the parasitic protozoa and higher eukaryotes. Biochem J.

[44] Naderer T, Ellis MA, Sernee MF, De Souza DP, Curtis J, Handman E, McConville MJ (2006). Virulence of Leishmania major in macrophages and mice requires the gluconeogenic enzyme fructose-1,6-bisphosphatase. Proc Natl Acad Sci U S A.

[45] McConville MJ, De Souza DP, Saunders EC, Pyke J, Naderer T, Ellis MA, Sernee FM, Ralton JE, Likic VA, Myler PJ, Fassel N (2008). Analysis of the Leishmania Metabolome. Leishmania After the Genome.

[46] Sacks DL (1989). Metacyclogenesis in Leishmania promastigotes. Exp Parasitol.

[47] Depledge DP, Evans KJ, Ivens AC, Aziz N, Maroof A, Kaye PM, Smith DF (2009). Comparative expression profiling of leishmania: modulation in gene expression between species and in different host genetic backgrounds. PLoS Negl Trop Dis.

[48] Anez N, Tang Y, Rojas A, Crisante G, Killick-Kendrick M, Killick-Kendrick R (2003). Detection of amastigote-like forms in the valve of Phlebotomus papatasi infected with Leishmania major. Mem Inst Oswaldo Cruz.

[49] Tang Y, Ward RD (1998). Sugar feeding and fluid destination control in the phlebotomine sandfly Lutzomyia longipalpis (Diptera: Psychodidae). Med Vet Entomol.

[50] McConville MJ, Mullin KA, Ilgoutz SC, Teasdale RD (2002). Secretory pathway of trypanosomatid parasites. Microbiol Mol Biol Rev.

[51] Suzuki E, Tanaka AK, Toledo MS, Takahashi HK, Straus AH (2002). Role of beta-D-galactofuranose in Leishmania major macrophage invasion. Infect Immun.

[52] Oppenheimer M, Valenciano AL, Sobrado P (2011). Biosynthesis of galactofuranose in kinetoplastids: novel therapeutic targets for treating leishmaniasis and chagas’ disease. Enzyme Res.

[53] Bolhassani A, Gholami E, Zahedifard F, Moradin N, Parsi P, Doustdari F, Seyed N, Papadopoulou B, Rafati S (2011). Leishmania major: protective capacity of DNA vaccine using amastin fused to HSV-1 VP22 and EGFP in BALB/c mice model. Exp Parasitol.

