# Supplementary material for: Stage-specific differential gene expression in Leishmania infantum: from the foregut of Phlebotomus perniciosus to the human phagocyte
Source: BMC Genomics. 2014 Oct 3;15(1):849. doi: 10.1186/1471-2164-15-849 (PMC4203910; doi:10.1186/1471-2164-15-849)
Supplement: Supplementary file 1 — Additional file 1: Primers and TaqMan-MGB probes used for qRT-PCR validation and the determination of differential expression in unresolved clones. Table S1. Sequences of qRT-PCR primers and probes. (PDF 212 KB) [file 12864_2014_6561_MOESM1_ESM.pdf]

TABLE S1. Sequences of qRT-PCR primers and probes.

Assay Name	Assay Mix Con.	Fw Primer Name	Forward Primer Seq.	Rv Primer Name	Reverse Primer Seq.	Rep. Name	Rep. Dye	Reporter Sequence	Reporter Quencher
PA010310	60x	AI89I9J_F	CGCTGCAGCTCTCTTTGAG	AI89I9J_R	TCAAGATTTCTGGAACGCTGAGT	AI89I9J_M	FAM	TCAGCACGTCATCCTT	NFQ
PA010330	60x	AIVI3C4_F	TCAACCTCCGCTGCATCAC	AIVI3C4_R	CGCGTCCTTCCGAGGAT	AIVI3C4_M	FAM	ATGCGCCCCACAGTAC	NFQ
PA030190	60x	AIWR1JC_F	CGGACGACTTCCAGAGCTT	AIWR1JC_R	GGCGATTTCGATGTACTTCTTATTCTTG	AIWR1JC_M	FAM	ATGTGCGCCGTCATCG	NFQ
PA030200	60x	AIX0ZPK_F	GTCAAACGACTCTGCGAATCAG	AIX0ZPK_R	ATCCGCGTCAAAGACATCCTT	AIX0ZPK_M	FAM	CTGGTCGGCACGCCCT	NFQ
PA060340	60x	AIS060O_F	CCGTGTTCCGGCTTGGA	AIS060O_R	TGCTGCCGCAGCTTTG	AIS060O_M	FAM	ACGCGCTCAAGTACG	NFQ
PA060350	60x	AIT946W_F	CATTGCGGAGAAGCAGTTGTAC	AIT946W_R	TGTCAGCATCGGTCACAATGTAG	AIT946W_M	FAM	AAGGTACGGCGAATTC	NFQ
PA061110	60x	AIMSF5K_F	GCCAGATGACGCCTTCCAT	AIMSF5K_R	TGCCCCGCTCGAGATC	AIMSF5K_M	FAM	CCTCGGCGACCTTC	NFQ
PA061320	60x	AI1RT78_F	AACGTGCACATTGGCATTCC	AI1RT78_R	CATCACAAAGCAGATTTCGTACACA	AI1RT78_M	FAM	CACGCCATGTATATTC	NFQ
PA080680	60x	AIPACH0_F	GTTCCTCTTCGGCTTCCTTTTG	AIPACH0_R	GGCAGAGCCAGCGGAA	AIPACH0_M	FAM	CAGCGCAGCAGTACAG	NFQ
PA080690	60x	AIQJAN8_F	GTTCCTCTTCGGCTTCCTTTTG	AIQJAN8_R	GGCAGAGCCAGCGGAA	AIQJAN8_M	FAM	CAGCGCAGCAGTACAG	NFQ
PA160470	60x	AI20SEF_F	ATCTGCGTCCGCTTCGA	AI20SEF_R	GTGCTCCTTCGCCTTGAATG	AI20SEF_M	FAM	ACGTCCGCAAGTCTC	NFQ
PA191490	60x	AIAAYFC_F	TCTGTGCAACCCTGCTCAAG	AIAAYFC_R	TGTGCCACACCCGATAAGAAAA	AIAAYFC_M	FAM	TTGACTGCACAAACTC	NFQ
PA200040	60x	AI1RT77_F	CGGAGGTGCAGCTGTACAA	AI1RT77_R	TGGCAGTGAGAACCTGTAGGTA	AI1RT77_M	FAM	ACGCACGCCGAAATG	NFQ
PA220600	60x	AIFAQ39_F	ATTGGAATGATTGCCGGTGGTA	AIFAQ39_R	GCGCGCTACCTGGTACA	AIFAQ39_M	FAM	TCGGCGTGATACCCG	NFQ
PA221370	60x	AIAAYFD_F	AGCGTCAGCCGCAACT	AIAAYFD_R	CTTCTCCAGCGCCTTCGA	AIAAYFD_M	FAM	CCAGCGCATCCTTCA	NFQ
PA230040	60x	AICSURS_F	CTGGCGGTGCACCCT	AICSURS_R	GTTGAGCTCGCCTGTTGTG	AICSURS_M	FAM	CCCACGGCGTACTCC	NFQ
PA230050	60x	AID1SX0_F	GCCGACTTTGAGAAGCTAAACAC	AID1SX0_R	CACGCCAGGTGAGAGTACAC	AID1SX0_M	FAM	ACACCGCAACAACC	NFQ
PA230060	60x	AIFAQ38_F	TCGATGGTCTTGACGTTGTGAAG	AIFAQ38_R	CGCAGTCAGACACCACGAT	AIFAQ38_M	FAM	CCAGTCGCTCAACTTT	NFQ
PA230630	60x	AIBJWLK_F	CTCCTTTCTACACATGGTCGACTTT	AIBJWLK_R	CACCGTTCGGCAGTTCCT	AIBJWLK_M	FAM	TTGCCGGTCGTATTCT	NFQ
PA242410	60x	AI70K3B_F	GATTCACCGTGATATCAAACCTGCTA	AI70K3B_R	CGCCGAAATCCGAGATCTTGA	AI70K3B_M	FAM	ACGTCCTGGCGAACAC	NFQ
PA250080	60x	AI6RMW3_F	GCATCCGCCTGCTTCAG	AI6RMW3_R	CTGCGGGAACGTGTTCATC	AI6RMW3_M	FAM	CCATGCGCCACCAGTC	NFQ
PA251850	60x	AI39QKN_F	AACATCTTCAAGAACTGCGTCTACT	AI39QKN_R	GACTGCACAGCACGTAACC	AI39QKN_M	FAM	TCCTTCGCCGCCTTC	NFQ
PA260380	60x	AIKAJS4_F	TGTCGGCTTCTTCCCAGATG	AIKAJS4_R	GTATCGGAGGTCCAGCATGTC	AIKAJS4_M	FAM	ATGCGCGAGGACTAC	NFQ
PA261620	60x	AI5IOQV_F	GCTGATTCGAAGAAGGACAACAAGA	AI5IOQV_R	CCTTGATGATCAGGCCAACCT	AI5IOQV_M	FAM	CTGGGTGACGACCTCC	NFQ
PA261680	60x	AIHSNGP_F	TCTGTACTTTTCTAGGCACCAGTTG	AIHSNGP_R	GCCCGTGAGCACAAAATGC	AIHSNGP_M	FAM	TCGCTGGTCACTTCAT	NFQ
PA300710	60x	AIGJPAH_F	GCAGGAGAAGCCGAAGCA	AIGJPAH_R	AGGAAGCGCCTTGTGTACTT	AIGJPAH_M	FAM	TCAGCGCACGGCCAC	NFQ
PA300830	60x	AIRR8UG_F	GCAAAGGCGATTCTTCCTCAAG	AIRR8UG_R	TGCTCTCGTGATCCTCTCCAT	AIRR8UG_M	FAM	CCCCGGCATATCAC	NFQ
PA310870	60x	AI0IV10_F	GCTGTGCTGAAGCTGATTCG	AI0IV10_R	CGAGCGAGTGCCCAGT	AI0IV10_M	FAM	CACCTACGAGATCCTC	NFQ
PA312330	60x	AIY9XVR_F	CCAACAATGCCGTATATCTCTGGTA	AIY9XVR_R	GCTGGTAGATGGCCTCGTTAAG	AIY9XVR_M	FAM	CCACTGCTCGATTATT	NFQ
PA312350	60x	AIN1EBS_F	GCGAAGGAAGAGCTGGACAA	AIN1EBS_R	CGAGCAGCACTGCATTACG	AIN1EBS_M	FAM	CCTCGGCCAAGATC	NFQ
PA313320	60x	AII1LMW_F	CGTGCTGAAGGCCTACGT	AII1LMW_R	CGCGCGTACTCGGTGTA	AII1LMW_M	FAM	CAGCGCACAATGTC	NFQ
PA322850	60x	AILJHZC_F	GTTCCTGCGCGTTGTGAA	AILJHZC_R	CCTTCGACATGTCCACGTTGTA	AILJHZC_M	FAM	CCACAAGCACTTCCTG	NFQ
PA340820	60x	AI20SEG_F	GCTGCGTGATTAGCGAGAAGAT	AI20SEG_R	GAAGAATCTCGTTAATGGCGGTAAG	AI20SEG_M	FAM	ACGGTGGCCTTAGTCC	NFQ
PA340830	60x	AI39QKO_F	AGTGCGCTAGCATCAACAAGAT	AI39QKO_R	ACGCCTTGAACAGCTTGATGT	AI39QKO_M	FAM	ACCGCCGCTTCACATC	NFQ
PA353930	60x	AI0IV1Z_F	AGGCGTTTCGTGTCTTCGA	AI0IV1Z_R	CGGCCACCGAGACTGT	AI0IV1Z_M	FAM	CCCGTCCAAGTCTTTG	NFQ
PA360560	60x	AI6RMW4_F	GGTCCCTGCCGAGGAG	AI6RMW4_R	GTTGACCCGACCATTTTCTACATG	AI6RMW4_M	FAM	CAGCAACGATTCTCG	NFQ
PA360570	60x	AI70K3C_F	CGAATCATTTTACTGCCTTCAGAGATG	AI70K3C_R	GTTCCTGTTGCACCGACTCT	AI70K3C_M	FAM	CAGCGCCCTCTCTGC	NFQ
PA361720	60x	AIGJPAG_F	GGTGGGTACGGCCAGAAG	AIGJPAG_R	TCGCCGCACTTGTAGCA	AIGJPAG_M	FAM	ATCGCCACCGTAGCCG	NFQ
PA361730	60x	AIHSNGO_F	TGGGAGCGCGTGCTT	AIHSNGO_R	GCCGCGACGGTGATG	AIHSNGO_M	FAM	CAGGCGGCACTCCAT	NFQ
PA362970	60x	AIX0ZPJ_F	GGGTGGAGAACGGCATGTC	AIX0ZPJ_R	CGGTGCTTCAGGAAGTTGTG	AIX0ZPJ_M	FAM	CTTGCTCCAGTCCTCG	NFQ
PA363170	60x	AIBJWLL_F	CACATTTACATCGAGGTCCTTCAGA	AIBJWLL_R	GCACGCCGCGTTGAC	AIBJWLL_M	FAM	CACGCGATTTTCTC	NFQ
PA363180	60x	AICSURT_F	CGTGCAGCCACCGATGA	AICSURT_R	GTGCGTGTCTTCGAGATCTCT	AICSURT_M	FAM	CTTGGCCGACAGCACC	NFQ
PA363190	60x	AID1SX1_F	GCCATTGTTGTGTACGAGCAA	AID1SX1_R	GCTGAGAGCGTACTCGTTGAG	AID1SX1_M	FAM	CCGACTGCATTATTTG	NFQ
PA363230	60x	AI5IOQW_F	CCAATCGTCAACATCCAACTTCTGT	AI5IOQW_R	CAGTGCCCAGCTGAACCA	AI5IOQW_M	FAM	ACGCTCGCCGATTGAG	NFQ
PA363750	60x	AI89I9K_F	GCGCGGGCGCTAAA	AI89I9K_R	GGCTCCACGGCAACGAT	AI89I9K_M	FAM	ATGACTGCCCATCTTC	NFQ
PA366370	60x	AIY9XVS_F	AAGAGCTGGAGTACCTCAAGGA	AIY9XVS_R	GAAGAGCTGCGTGATGATAAAGC	AIY9XVS_M	FAM	CCGGCATCCACCTTC	NFQ
