# Supplementary material for: Stage-specific differential gene expression in Leishmania infantum: from the foregut of Phlebotomus perniciosus to the human phagocyte
Source: BMC Genomics. 2014 Oct 3;15(1):849. doi: 10.1186/1471-2164-15-849 (PMC4203910; doi:10.1186/1471-2164-15-849)
Supplement: Supplementary file 2 — Additional file 2: Clones that map with UTRs or less than 5% of length of an ORF. Table S2. Clones that do not fulfill the criteria specified in section the Methods section, Microarray hybridization and analysis of data subsection. (DOC 205 KB) [file 12864_2014_6561_MOESM2_ESM.doc]

**Additional information about the Pro-Pper/Amas differential expression profile extracted from clones that overlap with UTRs or less than 5% of ORFs.**

The analysis of this clones has revealed that the genes coding for a DNA-binding protein, uracyl-DNA glycosylase, and a DEAD-box helicase protein are down-regulated in Pro-Pper with respect to amastigotes. The increase of the expression levels of the transcripts in suggests that DNA repair events take place in this intracellular stage. Indeed, the uracyl-DNA glycosylase is involved in base excision repair.

The up-regulation in Pro-Pper with respect to amastigotes of a signal aspartic peptidase, the putative RNA-binding protein of the DNA-directed RNA polymerase I subunit, the cyclophilin 3 and two phosphatases is additional evidence for the hypothesis of a descent of gene expression and intracellular signalling activities proposed in the article and in [23].

One of the proposals exposed in the article to explain that BLPL is up-regulated in Pro-Pper is the biosynthesis of fatty acids for the biosynthesis of lipid derivatives. This is supported by the up-regulation of a fatty acid desaturase and the phosphatidylethanolamine N-methyltransferase.

As for the cytoskeleton, a paraflagellar rod protein gene can be added to the list of genes up-regulated Pro-Pper in addition to actin, AIP and coronin.

**Table S2. Clones that do not fulfill the criteria specified in section the Methods section, Microarray hybridization and analysis of data subsection.** Most of these clones overlap with less than 5% of a given ORF or only with the 5'- or 3'-UTR region. A minority of clones do not overlap with any annotated gene. Fold changes (up-regulation if F > 2, over the dividing line, and down-regulation if F < -2, below the dividing line), base-two logarithmic scale F and their SD, p, e-values, clone definitions according to mapping outcomes a, b and c (see Methods section), overlapping with 5% ORF or UTRs only, annotation Ids. and annotated functions in the *L. infantum* genome sequence and the qRT-PCR outcomes.

| *Clone* | *F* | *Log_2_R ± S* | *p* | *e-value* | | *Def.* | *Overlaps* | *Annotation* | *Annotated gene function* |
| --- | --- | --- | --- | --- | --- | --- | --- | --- | --- |
|  |  |  |  | *Fw* | *Rv* |  |  |  |  |
| Lin10F7 | 3.67 | 1.9 ± 0.3 | 0.010 | 2e-34 | 4e-113 | b | 3'-UTR | LmjF.27.0960 | Hypothetical protein, conserved |
| Lin13G4 | 2.40 | 1.3 ± 0.4 | 0.038 | 0 | 0 | b | 5% ORF | LinJ.29.0990 | Signal aspartic peptidase, clan AD, family A22B, putative |
| Lin13H7 | 2.74 | 1.4 ± 0.5 | 0.035 | 0 | 0 | b |  |  |  |
| Lin16B12 | 5.86 | 2.5 ± 0.8 | 0.033 | 0 | 0 | b | 5% ORF | LinJ.25.1860 | Hypothetical protein, conserved |
| Lin16C6 | 3.13 | 1.6 ± 0.2 | 0.007 | 0 | 0 | a | 5% ORF | LinJ.20.0020 | Hypothetical protein, conserved |
|  |  |  |  |  |  |  |  | LinJ.20.0030 | Hypothetical protein, conserved |
| Lin21G5 | 7.61 | 2.9 ± 0.5 | 0.009 | 0 | 0 | b | 3'-UTR | LmjF.06.1350 | Hypothetical protein, unknown function |
| Lin29B8 | 3.14 | 1.6 ± 0.3 | 0.015 | 0 | 0 | b | 5% ORF | LinJ.35.1590 | Hypothetical protein, conserved |
| Lin31E7 | 3.60 | 1.8 ± 0.6 | 0.037 | - | 0 | c |  |  |  |
| Lin34D8 | 2.35 | 1.2 ± 0.3 | 0.023 | 0 | 0 | b | 5% ORF | LinJ.33.3160 | Hypothetical protein, unknown function |
| Lin38A2 | 6.10 | 2.6 ± 0.4 | 0.008 | 0 | 0 | a | 5'-UTR | LmjF.06.1300 | Hypothetical protein, conserved |
| Lin41C12 | 2.95 | 1.6 ± 0.5 | 0.028 | 0 | 0 | b | 5% ORF | LinJ.26.2300 | Hypothetical protein, conserved |
| Lin43B11 | 2.74 | 1.4 ± 0.3 | 0.013 | 1e-11 | 3e-61 | a |  |  |  |
| Lin43F7 | 4.91 | 2.3 ± 0.7 | 0.032 | 0 | 0 | b | 3'-UTR | LmjF.31.2290 | Phosphatidylethanolamine N-methyltransferase |
| Lin45A3 | 3.65 | 1.9 ± 0.4 | 0.015 | 0 | 0 | a | 5% ORF | LinJ.25.0090 | Hypothetical protein, conserved |
| Lin49A7 | 2.10 | 1.1 ± 0.3 | 0.034 | 0 | 0 | a | 5% ORF | LinJ.36.6360 | Hypothetical protein, conserved |
| Lin49B2 | 3.61 | 1.8 ± 0.5 | 0.020 | 0 | 0 | b | 5% ORF | LinJ.01.0290 | Hypothetical protein, conserved |
|  |  |  |  |  |  |  |  | LinJ.01.0300 | Hypothetical protein, conserved |
| Lin49B6 | 2.14 | 1.1 ± 0.2 | 0.007 | 0 | 0 | a | 5% ORF | LinJ.06.1310 | Hypothetical protein, conserved |
| Lin51A1 | 3.37 | 1.7 ± 0.7 | 0.047 | 9e-111 | 0 | c |  |  |  |
| Lin56B3 | 3.76 | 1.9 ± 0.7 | 0.041 | 0 | 0 | b | 5% ORF | LinJ.10.0930 | Hypothetical protein, conserved |
| Lin58C1 | 2.12 | 1.1 ± 0.2 | 0.013 | 2e-90 | 3e-90 | b | 5% ORF | LinJ.22.1370 | Hypothetical protein, unknown function |
| Lin58H2 | 3.67 | 1.9 ± 0.4 | 0.017 | 0 | 5e-106 | b | 5'-UTR | LmjF.20.1290 | Hypothetical protein, conserved |
| Lin65E7 | 2.26 | 1.2 ± 0.4 | 0.026 | 3e-104 | - | c |  |  |  |
| Lin70D9 | 3.85 | 1.9 ± 0.6 | 0.030 | 0 | 0 | b | 5% ORF | LinJ.24.0110 | Hypothetical predicted transmembrane protein |
| Lin74D2 | 3.09 | 1.6 ± 0.4 | 0.017 | 3e-36 | 1e-35 | a | 5'-UTR | LmjF.35.5240 | Hypothetical protein, unknown function |
| Lin75B1 | 7.37 | 2.9 ± 0.5 | 0.010 | 0 | 0 | a | 5'-UTR | LmjF.10.1320 | Fatty acid desaturase, putative |
| Lin76A8 | 7.16 | 2.8 ± 0.7 | 0.023 | 4e-11 | 0 | a | 5'-UTR | LmjF.31.0910 | Hypothetical protein, conserved |
| Lin76G12 | 9.94 | 3.3 ± 0.6 | 0.012 | 0 | 0 | a | 3'-UTR | LmjF.07.0010 | Ubiquitin-activating enzyme E1, putative |
| Lin77D4 | 9.49 | 3.2 ± 0.7 | 0.022 | 0 | 0 | a | 5'-UTR | LmjF.20.1290 | Hypothetical protein, conserved |
| Lin77F8 | 3.52 | 1.8 ± 0.5 | 0.022 | 3e-111 | - | c |  |  |  |
| Lin80C7 | 5.10 | 2.3 ± 0.7 | 0.028 | 0 | 0 | a | 5'-UTR | LmjF.23.0145 | Cyclophilin 3, putative |
| Lin81H1 | 3.74 | 1.9 ± 0.7 | 0.042 | 7e-173 | 0 | b | 5'-UTR | LmjF.23.0120 | Hypothetical protein, conserved |
| Lin86D3 | 12.0 | 3.6 ± 0.7 | 0.014 | 0 | 0 | b | 5'-UTR | LmjF.08.1100 | Hypothetical protein, conserved |
| Lin89D8 | 2.87 | 1.5 ± 0.4 | 0.023 | 0 | 0 | a | 5% ORF | LinJ.36.3240 | Hypothetical protein, conserved |
| Lin89F11 | 3.05 | 1.6 ± 0.3 | 0.010 | - | 4e-165 | c |  |  |  |
| Lin93D7 | 8.62 | 3.1 ± 0.7 | 0.015 | - | 0 | c |  |  |  |
| Lin95A12 | 3.31 | 1.7 ± 0.3 | 0.005 | 0 | 0 | b | 5'-UTR | LmjF.27.0240 | Kinetoplast-associated protein-like protein |
| Lin96B10 | 3.91 | 2.0 ± 0.1 | 0.001 | 0 | 0 | c |  |  |  |
| Lin100F12 | 2.84 | 1.5 ± 0.4 | 0.028 | 0 | 0 | a | 5% ORF | LinJ.30.3030 | Hypothetical protein, conserved |
| Lin104C3 | 4.45 | 2.1 ± 0.4 | 0.014 | 4e-57 | 0 | b | 5% ORF | LinJ.32.3100 | Nucleoside diphosphate kinase b |
| Lin106H3 | 2.23 | 1.2 ± 0.3 | 0.022 | 0 | 0 | b | 5% ORF | LinJ.30.3030 | Hypothetical protein, conserved |
| Lin106H5 | 2.39 | 1.3 ± 0.2 | 0.009 | 0 | 0 | b |  |  |  |
| Lin108H7 | 4.18 | 2.1 ± 0.1 | 0.001 | 0 | 0 | c |  |  |  |
| Lin122H12 | 5.11 | 2.3 ± 0.5 | 0.016 | 0 | 0 | b | 5'-UTR | LmjF.36.5870 | Paraflagellar rod component, putative |
| Lin123F5 | 2.17 | 1.1 ± 0.1 | 0.001 | 0 | 1e-159 | b | 5% ORF | LinJ.18.1290 | Hypothetical protein, conserved |
| Lin123F7 | 3.78 | 1.9 ± 0.4 | 0.018 | 0 | 0 | b | 5% ORF | LinJ.16.1030a | Hypothetical protein, unknown function |
| Lin127H6 | 3.10 | 1.6 ± 0.6 | 0.043 | 0 | 0 | b | 5% ORF | LinJ.05.0450 | Hypothetical protein, conserved |
| Lin128B11 | 3.06 | 1.6 ± 0.6 | 0.049 | 0 | 0 | a | 5% ORF | LinJ.18.1050 | 5-oxoprolinase, putative |
| Lin129C7 | 8.24 | 3.0 ± 0.6 | 0.013 | 3e-18 | 1e-17 | b | 5'-UTR | LmjF.13.1120 | DNA-directed RNA polymerase I subunit, putative RNA-binding protein |
| Lin129D7 | 4.19 | 2.1 ± 0.6 | 0.024 | 0 | 0 | b | 5% ORF | LinJ.36.4870 | Hypothetical protein, conserved |
| Lin130B7 | 3.44 | 1.8 ± 0.3 | 0.009 | 0 | 0 | a | 5% ORF | LinJ.23.0630 | Oxidoreductase-like protein |
| Lin131B1 | 8.64 | 3.1 ± 0.4 | 0.005 | 0 | 4e-159 | b | 5'-UTR | LmjF.31.1520 | Hypothetical protein, conserved |
| Lin131B10 | 2.23 | 1.2 ± 0.3 | 0.013 | 5e-106 | 2e-111 | b |  |  |  |
| Lin132B12 | 4.01 | 2.0 ± 0.5 | 0.023 | 0 | 0 | b | 5% ORF | LinJ.06.0810 | Hypothetical protein, unknown function |
| Lin135B3 | 4.74 | 2.2 ± 0.8 | 0.038 | 0 | 0 | a | 5% ORF | LinJ.30.0750 | Hypothetical protein, conserved |
| Lin138C1 | 6.11 | 2.6 ± 0.2 | 0.001 | 0 | 0 | a | 5% ORF | LinJ.24.1370 | Hypothetical protein, conserved |
| Lin139A5 | 7.01 | 2.8 ± 0.8 | 0.026 | 0 | 0 | b | 5% ORF | LinJ.11.0570 | Hypothetical protein, conserved |
| Lin142C8 | 5.97 | 2.6 ± 0.2 | 0.003 | 0 | 0 | a | 5% ORF | LinJ.21.0790 | Hypothetical protein, conserved |
| Lin142D3 | 3.27 | 1.7 ± 0.5 | 0.025 | 0 | 0 | a | 5% ORF | LinJ.22.0620 | Hypothetical protein, conserved |
| Lin157F10 | 2.83 | 1.5 ± 0.2 | 0.009 | 0 | 0 | b | 5% ORF | LinJ.31.2360 | Phosphatidylethanolamine N-methyltransferase |
| Lin161G4 | 5.36 | 2.4 ± 0.4 | 0.021 | 2e-154 | - | c |  |  |  |
| Lin162D7 | 6.68 | 2.3 ± 1.0 | 0.039 | 0 | 0 | a | 5'-UTR | LmjF.35.4640 | Hypothetical protein, conserved |
| Lin163E9 | 2.08 | 1.0 ± 0.4 | 0.046 | 0 | 1e-171 | b | 5% ORF | LinJ.27.1110 | Hypothetical protein, conserved |
| Lin168B2 | 2.78 | 1.5 ± 0.6 | 0.045 | 0 | 0 | b |  |  |  |
| Lin168F11 | 2.71 | 1.4 ± 0.3 | 0.018 | 0 | - | c |  |  |  |
| Lin169A11 | 4.37 | 2.1 ± 0.5 | 0.020 | 0 | 0 | a | 5% ORF | LinJ.23.0630 | Oxidoreductase-like protein |
| Lin169B2 | 2.00 | 1.0 ± 0.2 | 0.009 | 7e-59 | 7e-59 | b |  |  |  |
| Lin170A2 | 4.52 | 2.2 ± 0.3 | 0.007 | 0 | 0 | b | 3'-UTR | LmjF.29.0868 | Hypothetical protein, conserved |
| Lin170B2 | 3.72 | 1.9 ± 0.6 | 0.029 | 0 | 0 | a | 5% ORF | LinJ.31.3270 | Hypothetical protein, conserved |
| Lin172B9 | 4.42 | 2.1 ± 0.4 | 0.009 | 0 | 1e-11 | b | 5% ORF | LinJ.24.2340 | Hypothetical protein, conserved |
| Lin172E11 | 9.39 | 3.2 ± 0.3 | 0.002 | 7e-84 | 1e-94 | a | 5'-UTR | LmjF.36.0540 | Ubiquitin-like protein, putative |
| Lin173A1 | 2.23 | 1.1 ± 0.4 | 0.031 | 0 | 0 | b |  |  |  |
| Lin173B9 | 2.15 | 1.1 ± 0.4 | 0.038 | 0 | 0 | b | 5% ORF | LinJ.15.1410 | Actin-related protein arp3, putative |
| Lin188A5 | 3.52 | 1.8 ± 0.7 | 0.045 | 0 | 0 | b | 5'-UTR | LmjF.31.0080 | Amino acid permease 3, putative (AAP3) |
| Lin193F5 | 4.73 | 2.2 ± 0.4 | 0.010 | 0 | 0 | b | 5% ORF | LinJ.36.2090 | Serine/threonine protein phosphatase 2b, catalytic subunit A2, putative |
| Lin197D10 | 4.89 | 2.3 ± 0.3 | 0.005 | 0 | 3e-175 | b | 5% ORF | LinJ.19.0200 | ADP/ATP translocase 1, putative |
| Lin200D7 | 3.62 | 1.8 ± 0.4 | 0.014 | - | 0 | c |  |  |  |
| Lin202F9 | 3.49 | 1.8 ± 0.4 | 0.018 | 0 | 7e-167 | b | 5% ORF | LinJ.31.0570 | Hypothetical protein, conserved |
| Lin203H3 | 5.93 | 2.6 ± 0.7 | 0.026 | 0 | 0 | a | 3'-UTR | LmjF.32.2940 | Hypothetical protein, conserved |
| Lin210D4 | 2.99 | 1.6 ±0.1 | 0.001 | 0 | 0 | a | 5% ORF | LinJ.22.0690 | Hypothetical protein, conserved |
| Lin218B2 | 5.56 | 2.5 ± 0.1 | 0.000 | 3e-15 | 4e-14 | a | 5% ORF | LinJ.20.0970 | Protein kinase, putative |
| Lin219G10 | 4.18 | 2.1 ± 0.5 | 0.023 | 0 | 0 | b | 5'-UTR | LmjF.34.2750 | Methyltransferase-like protein |
| Lin223B1 | 6.32 | 2.7 ± 0.4 | 0.006 | 1e-116 | - | c |  |  |  |
| Lin225B11 | 5.98 | 2.6 ± 0.3 | 0.006 | 0 | 0 | a | 5% ORF | LinJ.24.1340 | Hypothetical protein, conserved |
| Lin228D4 | 4.90 | 2.3 ± 0.5 | 0.017 | 0 | 0 | a | 5% ORF | LinJ.19.0080 | Hypothetical protein, conserved |
| Lin229E11 | 3.07 | 1.6 ± 0.6 | 0.038 | 0 | 0 | b | 5'-UTR | LmjF.08.1100 | Hypothetical protein, conserved |
| Lin232H7 | 2.82 | 1.5 ± 0.3 | 0.011 | 0 | 0 | b | 5% ORF | LinJ.36.4870 | Hypothetical protein, conserved |
| Lin262A6 | 4.01 | 2.0 ± 0.4 | 0.012 | 0 | 0 | b |  |  |  |
| Lin267B9 | 3.19 | 1.7 ± 0.4 | 0.024 | 0 | 0 | b | 5% ORF | LinJ.36.0580 | Hypothetical protein, conserved |
| Lin274F11 | 3.88 | 1.9 ± 0.7 | 0.044 | 0 | 0 | a | 5'-UTR | LmjF.06.1300 | Hypothetical protein, conserved |
| Lin277D10 | 13.2 | 3.7 ± 0.2 | 0.001 | 0 | 0 | b | 3'-UTR | LmjF.26.2290 | Hypothetical protein, conserved |
| Lin280D11 | 7.73 | 2.9 ± 0.2 | 0.002 | 0 | 0 | b | 5% ORF | LinJ.25.2260 | Hypothetical protein, conserved |
| Lin287A5 | 2.08 | 1.1 ± 0.3 | 0.032 | 0 | 0 | b | 5% ORF | LinJ.33.1900 | Hypothetical protein, conserved |
|  |  |  |  |  |  |  |  | LinJ.33.1910 | Hypothetical protein, conserved |
| Lin288H7 | 2.86 | 1.5 ± 0.3 | 0.012 | 0 | 0 | b | 5% ORF | LinJ.34.2610 | Serine/threonine phosphatase, putative |
| Lin290F2 | 3.52 | 1.8 ± 0.3 | 0.012 | 0 | 0 | b | 5% ORF | LinJ.04.1250 | Actin |
| Lin292H10 | 2.15 | 1.1 ± 0.3 | 0.025 | 0 | 0 | a | 5% ORF | LinJ.08.0040 | Hypothetical protein, conserved |
| Lin294F5 | 17.1 | 4.1 ± 0.7 | 0.009 | 0 | 0 | b | 5'-UTR | LmjF.19.0210 | ADP/ATP translocase 1, putative (ANC2) |
| Lin299F5 | 2.4 | 1.3 ± 0.5 | 0.042 | 0 | 0 | b | 5% ORF | LinJ.09.0870 | Hypothetical protein, conserved |
| Lin309H8 | 5.10 | 2.3 ± 0.0 | 0.002 | 0 | 0 | b |  |  |  |
| Lin32C8 | -2.11 | -1.1 ± 0.3 | 0.027 | 8e-59 | 7e-59 | b | 5’-UTR | LmjF.35.0480 | Hypothetical protein, conserved |
| Lin34G5 | -3.72 | -1.9 ± 0.1 | 0.002 | 0 | 0 | b | 5’-UTR | LmjF.01.0790 | Hypothetical protein, conserved |
| Lin37B11 | -2.53 | -1.3 ± 0.5 | 0.044 | 2e-28 | 2e-25 | b | 3’-UTR | LmjF.25.0340 | Hypothetical protein, conserved |
| Lin38G10 | -2.66 | -1.4 ± 0.3 | 0.014 | 7e-59 | 7e-59 | b | 5’-UTR | LmjF.13.1010 | Hypothetical protein, conserved |
| Lin43B1 | -2.95 | -1.6 ± 0.3 | 0.013 | 5e-60 | 0 | b |  |  |  |
| Lin47F2 | -2.12 | -1.1 ± 0.4 | 0.040 | 7e-59 | 7e-59 | b | 3’-UTR | LmjF.17.0470 | Hypothetical protein, conserved |
| Lin51B4 | -2.35 | -1.2 ± 0.2 | 0.013 | 2e-56 | 2e-56 | b |  |  |  |
| Lin54B4 | -5.22 | -2.4 ± 0.8 | 0.032 | 5e-177 | 0 | b | 5'-UTR | LmjF.27.0050 | DEAD-box helicase-like protein |
| Lin54C8 | -2.17 | -1.1 ± 0.4 | 0.045 | 0 | 4e-116 | b |  |  |  |
| Lin55G12 | -2.09 | -1.1 ± 0.4 | 0.049 | 0 | 0 | a |  |  |  |
| Lin63A6 | -3.10 | -1.6 ± 0.5 | 0.032 | 0 | 0 | b | 3'-UTR | LmjF.21.0891 | Hypothetical protein |
| Lin65B12 | -2.39 | -1.3 ± 0.3 | 0.021 | 0 | 0 | b |  |  |  |
| Lin74B12 | -2.20 | -1.1 ± 0.4 | 0.043 | 7e-59 | 7e-59 | b |  |  |  |
| Lin81C8 | -3.78 | -1.9 ± 0.8 | 0.049 | 7e-59 | 7e-59 | b | 5'-UTR | LmjF.27.0050 | DEAD-box helicase-like protein |
| Lin89B4 | -2.12 | -1.1 ± 0.4 | 0.039 | 7e-59 | 7e-59 | b | 5'-UTR | LmjF.21.1510 | Hypothetical protein, conserved |
| Lin91D8 | -2.38 | -1.2 ± 0.4 | 0.032 | 7e-59 | 7e-59 | b |  |  |  |
| Lin95D8 | -3.93 | -1.97 ± 0.6 | 0.028 | 0 | 1e-14 | b | 5% ORF | LinJ.27.2470 | Hypothetical protein, conserved |
| Lin98G4 | -2.96 | -1.6 ± 0.6 | 0.041 | 0 | 7e-133 | b |  |  |  |
| Lin104E4 | -3.00 | -1.6 ± 0.3 | 0.015 | 0 | 0 | b | 3'-UTR | LmjF.36.3810 | Glycine synthase, putative |
| Lin109D1 | -3.06 | 1.6 ± 0.6 | 0.044 | 0 | 0 | b | 3'-UTR | LmjF.23.1390 | Hypothetical protein, conserved |
| Lin119B9 | -2.81 | -1.5 ± 0.3 | 0.010 | 0 | 0 | a | 5'-UTR | LmjF.11.0750 | Hypothetical protein, conserved |
| Lin128F5 | -8.08 | -3.0 ± 0.6 | 0.012 | 0 | 0 | b |  |  |  |
| Lin141H4 | -2.19 | -1.1 ± 0.1 | 0.006 | 6e-81 | 9e-84 | a | 3'-UTR | LmjF.03.0060 | Hypothetical protein, conserved |
| Lin156F2 | -2.88 | -1.5 ± 0.5 | 0.035 | 0 | 0 | b |  |  |  |
| Lin158B8 | -4.20 | -2.1 ± 0.4 | 0.015 | 0 | 0 | b | 3'-UTR | LmjF.24.1300 | DNA J domain protein, putative |
| Lin166F4 | -2.38 | -1.2 ± 0.4 | 0.036 | 7e-59 | 7e-59 | b |  |  |  |
| Lin166H5 | -2.32 | -1.2 ± 0.5 | 0.047 | 7e-59 | 7e-59 | b |  |  |  |
| Lin183B3 | -4.15 | -2.0 ± 0.8 | 0.049 | 7e-22 | 7e-22 | b | 5'-UTR | LmjF.27.0050 | DEAD-box helicase-like protein |
| Lin185F7 | -2.88 | -1.5 ± 0.6 | 0.044 | 7e-59 | 7e-59 | b |  |  |  |
| Lin189F2 | -2.97 | -1.6 ± 0.4 | 0.018 | 0 | 0 | b | 3'-UTR | LmjF.10.1230 | Hypothetical protein, conserved |
| Lin206H4 | -3.13 | -1.6 ± 0.3 | 0.014 | 0 | 0 | b | 5% ORF | LinJ.36.7210 | DNA-binding protein, putative |
| Lin208E10 | -3.10 | -1.6 ± 0.1 | 0.001 | 0 | 0 | a | 5'-UTR | LmjF.08.1100 | Hypothetical protein, conserved |
| Lin250H8 | -2.90 | -1.5 ± 0.6 | 0.043 | 7 e-59 | 7 e-59 | b |  |  |  |
| Lin256A6 | -6.31 | -2.7 ± 0.4 | 0.008 | 2e-87 | 1e-88 | a | 5% ORF | LinJ.18.0480 | Uracyl-DNA glycosylase, putative |
| Lin278B6 | -3.28 | -1.7 ± 0.4 | 0.022 | 7e-22 | 7e-22 | b |  |  |  |
