# Supplementary material for: Stage-specific differential gene expression in Leishmania infantum: from the foregut of Phlebotomus perniciosus to the human phagocyte
Source: BMC Genomics. 2014 Oct 3;15(1):849. doi: 10.1186/1471-2164-15-849 (PMC4203910; doi:10.1186/1471-2164-15-849)
Supplement: Supplementary file 3 — Additional file 3: Microarray controls. Table S3. The results of the Pro-Per/A cDNA-microarray hybridization analysis for positive and negative control spots. (DOCX 18 KB) [file 12864_2014_6561_MOESM3_ESM.docx]

##### Additional file 3. Microarray controls.

##### Table S3. Results of the Pro-Per/A cDNA-microarray hybridization analysis for positive and negative control spots. Pro-Pper/A fold changes (F) and standard deviations (SD) are detailed, as well as p-value, (α = 0,05). Absence of differential gene expression has been observed in positive controls (*p* ≥ 0,05), except for the genes A2 and p36, provided that they are up-regulated in amastigotes. Mean fluorescence intensity (FI) and the associated SD values are provided.

| ***Spot*** | **F ± SD** | ***P*** | **Positive control** |
| --- | --- | --- | --- |
| cLin79A1 | 1,0 ± 0,3 | 0,885 | *Li Polβ* |
| cLin79A2 | 1,0 ± 0,0 | 0,794 | *Li TopoII* |
| cLin79A3 | -1,9 ± 0,9 | 0,047 | *Li p36* |
| cLin79B1 | 1,1 ± 0,2 | 0,392 | *Li hsp70* |
| cLin79B2 | 1,0 ± 0,0 | 0,694 | *Ldo hsp70* |
| cLin79B3 | 1,0 ± 0,0 | 0,767 | *Lam hsp70* |
| cLin79C1 | 1,3 ± 0,5 | 0,273 | *Lma hsp70* |
| cLin79C2 | -1,8 ± 0,3 | 0,006 | *Li A2* |
| cLin79C3 | -1,5 ± 0,1 | 0,062 | *Ldo A2* |
| cLin79D1 | 1,2 ± 0,3 | 0,082 | *Li GAPDH* |
| cLin79D2 | 1,1 ± 0,2 | 0,674 | *Ldo GAPDH* |
| cLin79D3 | 1,1 ± 0,3 | 0,168 | *LigDNA* |
| cLin79H2 | 1,2 ± 0,3 | 0,201 | *HerringS DNA* |
| ***Spot*** | **Mean FI ± SD** | | **Negative control** |
| cLin79E1 | 402 ± 123 | | *Lfe nifA/hlyD* |
| cLin79E2 | 34 ± 23 | | *Lfe nifD/nifK* |
| cLin79E3 | 124 ± 32 | | *Lfe nifH* |
| cLin79F1 | 112 ±45 | | *Lfe nifS/nifU* |
| cLin79F2 | 183 ± 17 | | *Lfe nifX/nifB* |
| cLin79F3 | 112 ± 77 | | *Lfe nifH/nifD* |
| cLin79G1 | 221 ± 28 | | *Lfe nifE* |
| cLin79G2 | 154 ± 64 | | *Lfe nifV/HesB* |
| cLin79G3 | 22 ± 15 | | *Lfe nifV* |
| cLin79H1 | 83 ± 12 | | *Lfe nifW/Bgene* |
| cLin79H3 | 58 ± 21 | | *1XSSC* |
|  |  | |  |
