# Supplementary material for: Stage-specific differential gene expression in Leishmania infantum: from the foregut of Phlebotomus perniciosus to the human phagocyte
Source: BMC Genomics. 2014 Oct 3;15(1):849. doi: 10.1186/1471-2164-15-849 (PMC4203910; doi:10.1186/1471-2164-15-849)
Supplement: Supplementary file 4 — Additional file 4: Hypothetical proteins. Table S4. Hypothetical proteins up-regulated in Pro-Pper/A. Table S5. Hypothetical proteins down-regulated in Pro-Pper/A. (DOC 168 KB) [file 12864_2014_6561_MOESM4_ESM.doc]

**Table S4. Hypothetical proteins up-regulated in Pper/A.**

| *Clone* | *F* | *Log_2_R ± S* | *p* | *e-value* | | *Def.* | *Annotation* | *Annotated gene function* |
| --- | --- | --- | --- | --- | --- | --- | --- | --- |
|  |  |  |  | *Fw* | *Rv* |  |  |  |
| Lin9F8 | 2.60 | 1.4 ± 0.5 | 0.040 | 0 | 0 | b | LinJ.33.3100 | Hypothetical protein, conserved |
| Lin16A12 | 2.21 | 1.1 ± 0.2 | 0.008 | 0 | 0 | b | LinJ.16.1290 | Hypothetical protein, unknown function |
|  |  |  |  |  |  |  | LinJ.16.1300 | Hypothetical protein, conserved |
|  |  |  |  |  |  |  | LinJ.16.1310 | Hypothetical protein, conserved |
| Lin26A1 | 4.01 | 2.0 ± 0.4 | 0.012 | 0 | 0 | b | LinJ.25.0090 | Hypothetical protein, conserved |
|  |  |  |  |  |  |  | LinJ.25.0100 | Hypothetical protein, conserved |
| Lin28F4 | 2.95 | 1.6 ± 0.3 | 0.011 | 0 | 0 | a | LinJ.35.3520 | Hypothetical protein, conserved |
| Lin35B6 | 2.34 | 1.2 ± 0.3 | 0.026 | 0 | 0 | b | LinJ.28.0740 | Hypothetical protein, conserved |
| Lin43A9 | 5.93 | 2.5 ± 0.7 | 0.023 | 0 | 0 | b | LinJ.33.0660 | Hypothetical protein, conserved |
|  |  |  |  |  |  |  | LinJ.33.0670 | Hypothetical protein, conserved |
|  |  |  |  |  |  |  | LinJ.33.0680 | Hypothetical protein, conserved |
| Lin43F10 | 2.31 | 1.2 ± 0.3 | 0.015 | 0 | 0 | b | LinJ.35.4700 | Hypothetical protein, conserved |
| Lin57F10 | 3.16 | 1.7 ± 0.2 | 0.006 | 8e-28 | 3e-21 | b | LinJ.31.2730 | Hypothetical protein, unknown function |
| Lin64A7 | 2.19 | 1.1 ± 0.4 | 0.035 | 0 | 0 | b | LinJ.09.0590 | Hypothetical protein, unknown function |
| Lin74F12 | 3.55 | 1.8 ± 0.1 | 0.001 | 0 | 0 | a | LinJ.36.0510 | Hypothetical protein, conserved |
|  |  |  |  |  |  |  | LinJ.36.0520 | Hypothetical protein, conserved |
| Lin76F12 | 2.63 | 1.4 ± 0.5 | 0.042 | 0 | 7e-25 | b | LinJ.34.3040 | Hypothetical protein, conserved |
| Lin77G12 | 3.24 | 1.7 ± 0.6 | 0.042 | 0 | 0 | b | LinJ.32.1520 | Hypothetical protein, conserved |
| Lin80G4 | 3.43 | 1.8 ± 0.3 | 0.012 | 0 | 0 | a | LinJ.12.0500 | Hypothetical protein, conserved |
| Lin81H4 | 5.28 | 2.4 ± 0.6 | 0.021 | 0 | 0 | a | LinJ.09.1610 | Hypothetical protein, conserved |
|  |  |  |  |  |  |  | LinJ.09.1620 | Hypothetical protein, conserved |
| Lin85E11 | 2.80 | 1.5 ± 0.5 | 0.034 | 4e-162 | 0 | b | LinJ.23.0870 | Hypothetical protein, conserved |
| Lin94G2 | 3.60 | 1.8 ± 0.7 | 0.044 | 0 | 0 | a | LinJ.23.0870 | Hypothetical protein, conserved |
| Lin96H11 | 2.77 | 1.5 ± 0.4 | 0.020 | 4e-153 | 9e-148 | a | LinJ.26.1570 | Hypothetical protein, unknown function |
| Lin97E2 | 7.32 | 2.9 ± 0.5 | 0.012 | 0 | 0 | b | LinJ.23.0010 | Hypothetical protein, conserved |
| Lin100H5 | 2.14 | 1.1 ± 0.3 | 0.034 | 0 | 0 | b | LinJ.27.0490 | Hypothetical protein, conserved |
| Lin106F1 | 5.78 | 2.5 ± 0.3 | 0.006 | 0 | 0 | b | LinJ.35.3970 | Hypothetical protein, conserved |
| Lin106G8 | 5.94 | 2.6 ± 0.6 | 0.021 | 0 | 0 | b | LinJ.24.2430 | Hypothetical protein, conserved |
| Lin107G9 | 8.76 | 3.1 ± 0.6 | 0.013 | 0 | 0 | b | LinJ.13.1430 | Hypothetical protein, conserved |
|  |  |  |  |  |  |  | LinJ.13.1440 | Hypothetical protein, unknown function |
| Lin110B6 | 4.16 | 2.1 ± 0.4 | 0.015 | 0 | 0 | b | LinJ.36.5200 | Hypothetical protein, conserved |
| Lin112D11 | 2.32 | 1.2 ± 0.4 | 0.043 | 1e-69 | 0 | b | LinJ.31.2430 | Hypothetical protein, conserved |
| Lin119D12 | 2.06 | 1.0 ± 0.4 | 0.037 | 0 | 0 | b | LinJ.31.2450 | Hypothetical protein, conserved |
| Lin123C11 | 2.68 | 1.4 ± 0.5 | 0.040 | 0 | 0 | b | LinJ.15.0970 | Hypothetical protein, conserved |
| Lin128C11 | 4.47 | 2.2 ± 0.0 | 0.013 | 3e-166 | 7e-167 | b | LinJ.26.2300 | Hypothetical protein, conserved |
| Lin129E3 | 3.64 | 1.9 ± 0.2 | 0.003 | 0 | 0 | a | LinJ.36.6610 | Hypothetical protein, conserved |
|  |  |  |  |  |  |  | LinJ.36.6620 | Hypothetical protein, conserved |
| Lin131F11 | 4.00 | 2.0 ± 0.7 | 0.042 | 0 | 0 | a | LinJ.34.0040 | Hypothetical protein, conserved |
|  |  |  |  |  |  |  | LinJ.34.0050 | Hypothetical protein, conserved |
| Lin133F9 | 3.00 | 1.6 ± 0.5 | 0.036 | 0 | 0 | b | LinJ.35.5310 | Hypothetical protein, conserved |
| Lin134A3 | 2.61 | 1.4 ± 0.3 | 0.017 | 0 | 0 | b | LinJ.04.0630 | Hypothetical protein, conserved |
|  |  |  |  |  |  |  | LinJ.04.0640 | Hypothetical protein, conserved |
| Lin134B10 | 2.97 | 1.6 ± 0.5 | 0.035 | 0 | 0 | b | LinJ.31.2300 | Hypothetical protein, unknown function |
| Lin134B3 | 2.28 | 1.2 ± 0.3 | 0.027 | 0 | 0 | a | LinJ.10.1340 | Hypothetical protein |
|  |  |  |  |  |  |  | LinJ.10.1350 | Hypothetical protein |
| Lin135B3 | 4.74 | 2.2 ± 0.8 | 0.038 | 0 | 0 | a | LinJ.09.1630 | Hypothetical protein, conserved |
|  |  |  |  |  |  |  | LinJ.09.1640 | Hypothetical protein, conserved |
| Lin142B1 | 2.33 | 1.2 ± 0.4 | 0.033 | 0 | 0 | b | LinJ.25.0100 | Hypothetical protein, conserved |
|  |  |  |  |  |  |  | LinJ.25.0110 | Hypothetical protein, conserved |
| Lin148B12 | 2.38 | 1.2 ± 0.2 | 0.009 | 0 | 0 | b | LinJ.09.1060 | Hypothetical protein, conserved |
| Lin149D5 | 2.45 | 1.3 ± 0.4 | 0.031 | 0 | 0 | b | LinJ.32.2530 | Hypothetical protein, conserved |
| Lin158A9 | 2.54 | 1.3 ± 0.2 | 0.005 | 0 | 0 | b | LinJ.30.2310 | Hypothetical protein, conserved |
|  |  |  |  |  |  |  | LinJ.30.2320 | Hypothetical protein, conserved |
|  |  |  |  |  |  |  | LinJ.30.2330 | Hypothetical protein, conserved |
| Lin168H2 | 2.64 | 1.4 ± 0.2 | 0.008 | 0 | 0 | b | LinJ.06.0050 | Hypothetical protein, conserved |
| Lin184C3 | 4.76 | 2.2 ± 0.5 | 0.015 | 0 | 0 | b | LinJ.34.0310 | Hypothetical protein, conserved |
| Lin204D8 | 8.22 | 3.0 ± 0.9 | 0.026 | 0 | 0 | a | LinJ.33.0650 | Hypothetical protein, conserved |
|  |  |  |  |  |  |  | LinJ.33.0660 | Hypothetical protein, conserved |
|  |  |  |  |  |  |  | LinJ.33.0670 | Hypothetical protein, conserved |
| Lin208D12 | 4.10 | 2.0 ± 0.4 | 0.011 | 0 | 0 | a | LinJ.35.3960 | Hypothetical protein, conserved |
|  |  |  |  |  |  |  | LinJ.35.3970 | Hypothetical protein, conserved |
| Lin210D4 | 3.23 | 1.9 ± 0.1 | 0.002 | 0 | 7e-167 | b | LinJ.22.0620 | Hypothetical protein, conserved |
| Lin229C7 | 5.00 | 2.3 ± 0.8 | 0.038 | 0 | 0 | b | LinJ.29.0760 | Hypothetical protein, conserved |
| Lin233F4 | 3.52 | 1.8 ± 0.6 | 0.040 | 0 | 0 | b | LinJ.30.3590 | Hypothetical protein, conserved |
| Lin282G9 | 7.32 | 2.9 ± 0.8 | 0.023 | 6e-44 | 3e-58 | a | LinJ.29.1260 | Hypothetical protein, conserved |
| Lin284D11 | 6.90 | 2.8 ± 0.5 | 0.010 | 0 | 0 | b | LinJ.27.0130 | Hypothetical protein, conserved |
|  |  |  |  |  |  |  | LinJ.27.0140 | Hypothetical protein, conserved |
| Lin280D11 | 7.73 | 2.9 ± 0.2 | 0.002 | 0 | 0 | a | LinJ.23.1370 | Hypothetical protein, conserved |
|  |  |  |  |  |  |  | LinJ.23.1380 | Hypothetical protein, conserved |

**Table S5. Hypothetical proteins down-regulated in Pper/A.**

| *Clon* | *F* | *Log_2_R ± S* | *p* | *Valor e* | | *Def.* | *Anotación* | *Función génica anotada* |
| --- | --- | --- | --- | --- | --- | --- | --- | --- |
|  |  |  |  | *Fw* | *Rv* |  |  |  |
| Lin31F6 | -2.10 | -1.1 ± 0.2 | 0.019 | 9e-74 | 3e-141 | b | LinJ.35.0180 | Hypothetical protein, conserved |
| Lin36C2 | -2.19 | -1.2 ± 0.4 | 0.029 | 0 | 0 | b | LinJ.33.2470 | Hypothetical protein, conserved |
| Lin47A4 | -2.03 | -1.0 ± 0.3 | 0.033 | 0 | 0 | a | LinJ.24.1430 | Hypothetical protein, conserved |
| Lin79B10 | -2.55 | -1.3 ± 0.3 | 0.021 | 0 | 0 | b | LinJ.35.2580 | Hypothetical protein, unknown function |
| Lin90H3 | -2.24 | -1.2 ± 0.5 | 0.049 | 0 | 0 | a | LinJ.19.1540 | Hypothetical protein, conserved |
| Lin94E7 | -2.18 | -1.1 ± 0.3 | 0.030 | 0 | 0 | b | LinJ.16.1070 | Hypothetical protein, conserved |
| Lin99E11 | -2.35 | -1.2 ± 0.5 | 0.043 | 0 | 0 | a | LinJ.30.3250 | Hypothetical protein, conserved |
| Lin100H12 | -2.00 | -1.0 ± 0.3 | 0.029 | 0 | 0 | a | LinJ.23.1880 | Hypothetical protein, conserved |
|  |  |  |  |  |  |  | LinJ.23.1890 | Hypothetical protein, unknown function |
| Lin104C12 | -2.33 | -1.2 ± 0.0 | 0.045 | 0 | 0 | b | LinJ.18.0390 | Hypothetical protein, conserved |
| Lin111G5 | -2.63 | -1.4 ± 0.5 | 0.036 | 6e-140 | 0 | b | LinJ.12.0090 | Hypothetical protein, conserved |
| Lin120D4 | -2.26 | -1.2 ± 0.2 | 0.014 | 0 | 0 | b | LinJ.36.2960 | Hypothetical protein, conserved |
| Lin121G9 | -2.22 | -1.1 ± 0.2 | 0.013 | 0 | 0 | a | LinJ.28.0510 | Hypothetical protein, unknown function |
|  |  |  |  |  |  |  | LinJ.28.0520 | Hypothetical protein, conserved |
| Lin125A5 | -2.17 | -1.1 ± 0.3 | 0.030 | 0 | 0 | b | LinJ.32.2410 | Hypothetical protein, conserved |
| Lin142F8 | -2.38 | -1.2 ± 0.2 | 0.018 | 0 | 0 | a | LinJ.29.2340 | Hypothetical protein, conserved |
| Lin148E3 | -2.26 | -1.2 ± 0.3 | 0.003 | 0 | 3e-92 | a | LinJ.11.0920 | Hypothetical protein, conserved |
| Lin165B10 | -2.94 | 1.6 ± 0.5 | 0.034 | 0 | 0 | a | LinJ.30.1620 | Hypothetical protein, conserved |
| Lin171B2 | -3.72 | -1.9 ± 0.7 | 0.045 | 2e-176 | 0 | b | LinJ.24.0560 | Hypothetical protein, conserved |
| Lin181H11 | -4.21 | -2.1 ± 0.8 | 0.047 | 0 | 0 | a | LinJ.18.0720 | Hypothetical protein, conserved |
| Lin206E8 | -2.84 | -1.5 ± 0.4 | 0.024 | 0 | 0 | b | LinJ.36.0900 | Hypothetical protein, conserved |
| Lin206H3 | -2.53 | -1.3 ± 0.1 | 0.004 | 0 | 0 | b | LinJ.10.1370 | Hypothetical protein, conserved |
| Lin209A6 | -2.39 | -1.2 ± 0.3 | 0.023 | 0 | 0 | b | LinJ.22.1070 | Hypothetical protein, conserved |
| Lin210F2 | -2.67 | -1.4 ± 0.3 | 0.021 | 0 | 0 | b | LinJ.32.1100 | Hypothetical protein, conserved |
| Lin224H11 | -2.22 | -1.1 ± 0.4 | 0.045 | 0 | 0 | a | LinJ.16.1620 | Hypothetical protein, conserved |
