# Supplementary material for: Stage-specific differential gene expression in Leishmania infantum: from the foregut of Phlebotomus perniciosus to the human phagocyte
Source: BMC Genomics. 2014 Oct 3;15(1):849. doi: 10.1186/1471-2164-15-849 (PMC4203910; doi:10.1186/1471-2164-15-849)
Supplement: Supplementary file 5 — Additional file 5: Overview of the MEV clustering analysis. Figure S1. Profile of clusters of genes differentially regulated in amastigotes. (PPTX 310 KB) [file 12864_2014_6561_MOESM5_ESM.pptx]

## Slide 1
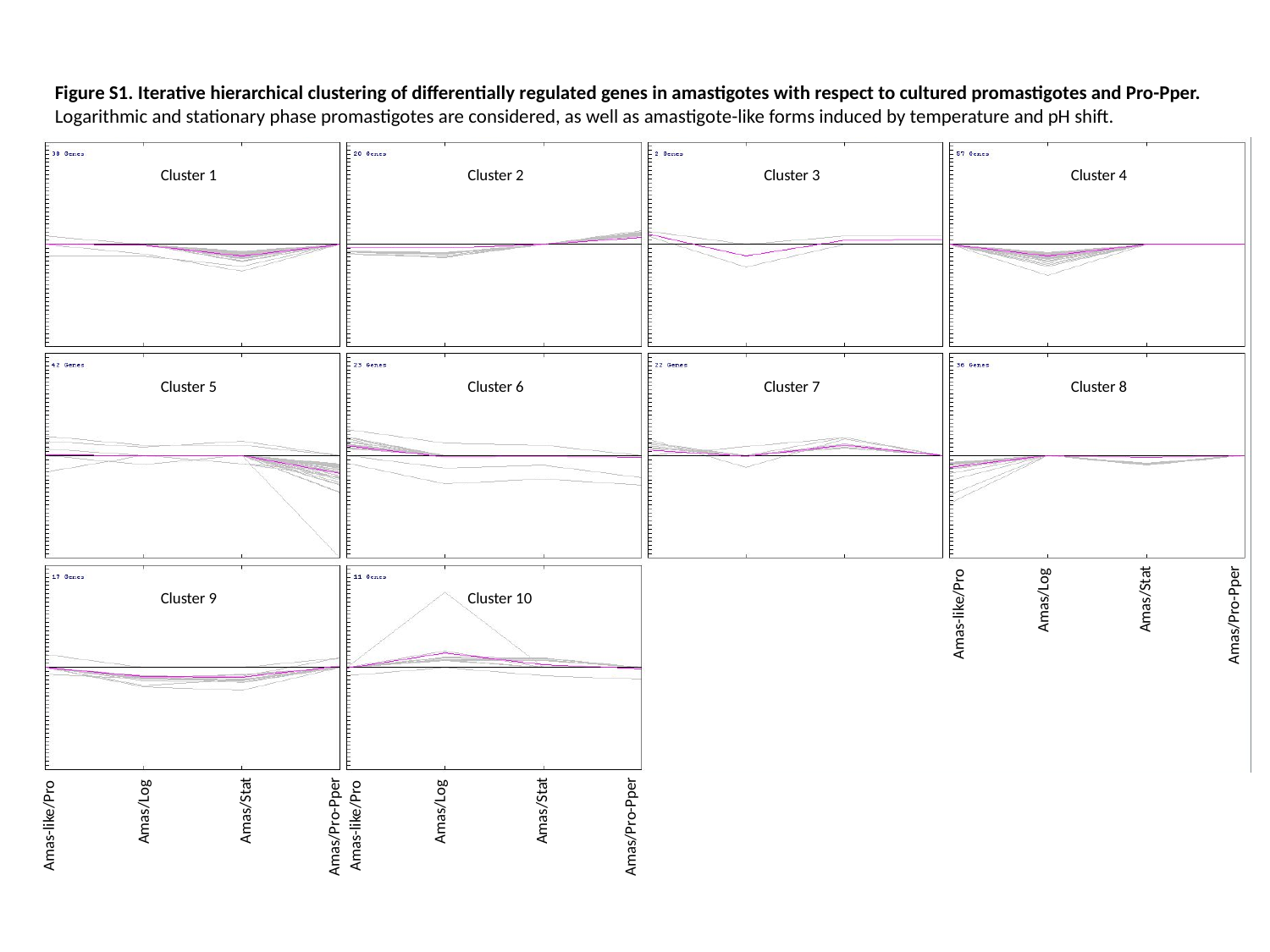

Figure S1. Iterative hierarchical clustering of differentially regulated genes in amastigotes with respect to cultured promastigotes and Pro-Pper. Logarithmic and stationary phase promastigotes are considered, as well as amastigote-like forms induced by temperature and pH shift.
Cluster 1
Cluster 2
Cluster 3
Cluster 4
Cluster 5
Cluster 6
Cluster 7
Cluster 8
Cluster 9
Cluster 10
Amas/Log
Amas/Stat
Amas-like/Pro
Amas/Pro-Pper
Amas/Log
Amas/Stat
Amas/Log
Amas/Stat
Amas-like/Pro
Amas-like/Pro
Amas/Pro-Pper
Amas/Pro-Pper
